# ATP11B inhibits breast cancer metastasis in a mouse model by suppressing externalization of nonapoptotic phosphatidylserine

**DOI:** 10.1172/JCI149473

**Published:** 2022-03-01

**Authors:** Jun Xu, Sek Man Su, Xin Zhang, Un In Chan, Ragini Adhav, Xiaodong Shu, Jianlin Liu, Jianjie Li, Lihua Mo, Yuqing Wang, Tingting An, Josh Haipeng Lei, Kai Miao, Chu-Xia Deng, Xiaoling Xu

**Affiliations:** 1 Cancer Centre, Faculty of Health Sciences,; 2 Centre for Precision Medicine Research and Training, Faculty of Health Sciences, and; 3 Ministry of Education Frontiers Science Center for Precision Oncology, University of Macau, Macau, China.

**Keywords:** Oncology, Breast cancer, Cancer immunotherapy, Tumor suppressors

## Abstract

Cancer metastasis is the cause of the majority of cancer-related deaths. In this study, we demonstrated that no expression or low expression of ATP11B in conjunction with high expression of PTDSS2, which was negatively regulated by BRCA1, markedly accelerates tumor metastasis. Further analysis revealed that cells with low ATP11B expression and high PTDSS2 expression (ATP11B^lo^PTDSS2^hi^ cells) were associated with poor prognosis and enhanced metastasis in breast cancer patients in general. Mechanistically, an ATP11B^lo^PTDSS2^hi^ phenotype was associated with increased levels of nonapoptotic phosphatidylserine (PS) on the outer leaflet of the cell membrane. This PS increase serves as a global immunosuppressive signal to promote breast cancer metastasis through an enriched tumor microenvironment with the accumulation of myeloid-derived suppressor cells and reduced activity of cytotoxic T cells. The metastatic processes associated with ATP11B^lo^PTDSS2^hi^ cancer cells can be effectively overcome by changing the expression phenotype to ATP11B^hi^PTDSS2^lo^ through a combination of anti-PS antibody with either paclitaxel or docetaxel. Thus, blocking the ATP11B^lo^PTDSS2^hi^ axis provides a new selective therapeutic strategy to prevent metastasis in breast cancer patients.

## Graphical abstract

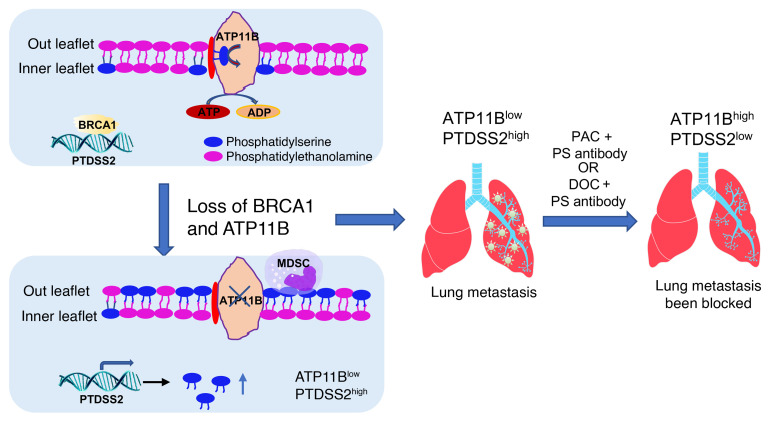

## Introduction

Breast cancer is the most common type of cancer and the second leading cause of cancer mortality among women in the world (1, 2). While the majority of breast cancers occur sporadically in women without a clear family history, approximately 5%–10% of breast cancers are hereditary and are caused by gene mutations passed through the germline, including breast cancer–associated gene 1 (*BRCA1*), *BRCA2*, *p53*, and *ATM* (3). Germline mutations of *BRCA1* have been found to contribute to approximately 25%–40% of all familial breast cancer cases and most familial ovarian cancer cases (4, 5). Approximately 60% of *BRCA1*-associated breast cancers are triple negative (TNBC), i.e., negative for estrogen receptor (*ER*), progesterone receptor (*PR*), and human epidermal growth factor receptor 2 (*HER2*); these are very aggressive and high-grade cancers with a poor prognosis (6, 7).

To study the biology of *BRCA1*-associated cancers, we previously created a mouse model carrying a mammary tissue–specific deletion of the full-length form of *BRCA1* using a Cre-*loxP* approach (8). We observed that the *BRCA1*-MT mammary epithelium carrying the mutant strain *Brca1^Co/Co^ MMTV-Cre* (*Brca1*-MSK) shows an initial growth disadvantage and increased apoptosis rate accompanied by genome instability. Moreover, approximately 25% of these mice eventually developed mammary tumors by approximately 1.5 years of age, and the tumorigenesis was markedly enhanced when a heterozygous mutation of p53 was introduced into these mice (*Brca1*-MSK *p53^+/–^*) (8–10). Our recent studies on this strain of mice revealed that *BRCA1* deficiency triggers alterations in multiple events, including mutations in potential tumor metastasis suppressors, an altered epithelial-mesenchymal transition, impaired mitophagy, and increased inflammasome activation, all of which may promote mammary tumor metastasis (11–13). Several studies also indicated that human patients with *BRCA1*-deficient breast cancer experienced more frequent locoregional recurrence and multiple-organ metastasis, especially in the lung and distant lymph nodes, than patients with sporadic breast cancers (14, 15).

Because cancer metastasis is the cause of death in 90% of deaths from cancer, there is an urgent need to understand the mechanisms underlying metastasis, especially the driving factors (16). It is generally believed that cancer metastasis involves a multistep process driven by metastasis-associated genes or metastasis-specific drivers. However, many recent high-throughput studies using paired primary and metastatic cancers have not led to the identification of genes mutated exclusively in metastatic cancers; in contrast, the majority of the genes found in metastatic cancers are also present in primary cancers (17, 18). Recent studies highlighted that the tumor microenvironment (TME) plays very important roles in tumorigenesis and tumor metastasis (19, 20). Mutations that cause altered gene expression in tumor cells can educate surrounding immune cells to establish or strengthen the TME for the growth and metastasis of tumor cells (21). Different mutations may direct different education programs through different molecular mechanisms (22). In this regard, it has been shown that phosphatidylserine (PS) is an evolutionarily conserved antiinflammatory and immunosuppressive signal of cancer and that PS-related signaling is highly dysregulated in the TME (23, 24); yet the driving force that maintains PS on the outer leaflet of the cell membrane as a nonapoptotic signal and promotes cancer metastasis remains elusive.

In this study, we performed CRISPR/Cas9–mediated genome-wide screening and identified *ATP11b* as a potent metastatic suppressor in mice carrying mammary-specific knockout of *Brca1*. Our study demonstrates that low levels of *ATP11B* expression and high levels of *PTDSS2* expression promote breast cancer metastasis by increasing nonapoptotic PS populations on the outer leaflet of the cell membrane. This finding may facilitate the development of a selective treatment strategy for breast cancer patients to prevent cancer metastasis.

## Results

### Identification of metastatic repressors using CRISPR/Cas9-mediated genome-wide screening.

**Figure 1:**
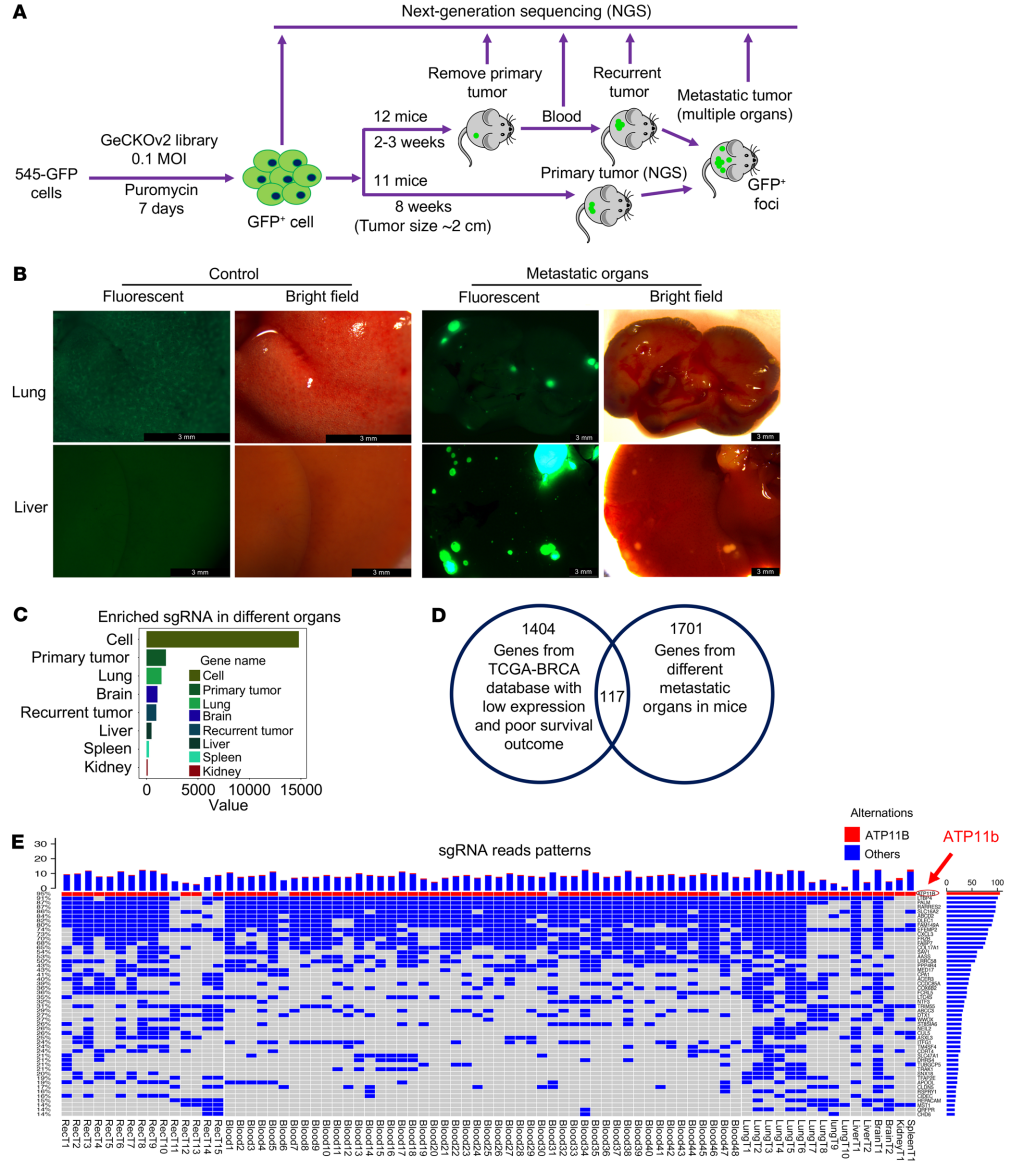
Genome-wide screening of tumor metastatic repressors with a CRISPR/Cas9 sgRNA library in *Brca1*-MT 545 cells. (**A**) Workflow of screening tumor metastatic repressors using CRISPR/Cas9 (GeCKOv2 sgRNA) library–infected 545 cells, which were implanted into the mammary fat pad of nude mice for tumor formation. Samples from the primary tumors, blood samples, recurrent tumors, and metastatic tumors were harvested for next-generation sequencing as indicated (*n* = 23 mice). (**B**) Representative images of lungs and livers from nude mice implanted with 545 cells only (control, *n* = 16) or 545 cells infected with the GeCKOv2 sgRNA library. Scale bars: 3 mm.(**C**) Enriched sgRNA reads count (10 and more than 10 for every sample) from primary tumors (*n* = 32), recurrent tumors (*n* = 15), and metastatic tumors (*n* = 16) from the lung, brain, liver, spleen, and kidney compared with cells infected with the GeCKOv2 sgRNA library, as plotted by ggplot2 (https://cran.r-project.org/web/packages/ggplot2/index.html). (**D**) A total of 117 potential human homologous tumor suppressors were identified from a Venn diagram analysis of 1521 potential human tumor suppressors with 1818 potential tumor metastatic repressors obtained from GeCKOv2 sgRNA library screening in vivo. A total of 1521 genes were obtained from 9386 overlapping genes that had low expression compared with that of healthy donors, with 2881 genes related to poor survival outcomes (*P* < 0.05) when their expression was lower than that of the same genes in the TCGA-BRCA database. (**E**) The top 50 human homologous genes with sgRNA reads equal to or greater than 10 were found in all 15 recurrent tumors (RecT), 48 blood samples, and 16 metastatic organ–specific genes, including the lung (LungT), liver (LiverT), brain (BrainT), kidney (KidneyT), and spleen (SpleenT), in mice by Oncoplot (https://github.com/jokergoo/ComplexHeatmap).

To gain a comprehensive understanding of tumor metastasis associated with *Brca1* deficiency, we analyzed a cohort of 207 tumor-bearing *Brca1*-MSK mice and found that 47 mice (22%) developed metastasis in multiple organs (Supplemental Figure 1A). To identify metastatic suppressors, we used *Brca1*-mutant (*Brca1*-MT) 545 cells, which were isolated from a primary mammary tumor of a *Brca1*-MSK mouse (8). These cells were labeled with luciferase-GFP and implanted orthotopically into the mammary fat pad of nude mice. No metastasis was detected in the lung when the primary tumors reached approximately 2 cm in diameter 8 weeks after implantation into the fat pad. Next, we infected the 545-GFP cells with a CRISPR/Cas9 (GeCKOv2 sgRNA) library containing 130,209 sgRNAs that target 20,611 genes (6 sgRNAs per gene). After selection with puromycin for 7 days, the cells were implanted into the mammary fat pad of 23 nude mice, and samples including primary tumors, recurrent tumors, blood, and metastatic tumors were harvested for next-generation sequencing following the procedure shown in Figure 1A. We detected metastatic signals in the lungs of all mice that were implanted with 545-GFP-GeCKOv2 cells, and in several other organs, including the brain, liver, spleen, and/or kidney. We also found that upon the removal of the primary tumor, the mice exhibited, on average, 3-fold more metastatic nodules in the lung and some other organs, which is consistent with the general observation that the removal of primary tumors enhances tumor metastasis. As a control, we also implanted the parental 545 cells into the mammary fat pad of 16 mice, and we did not observe metastatic GFP signals when their primary tumors reached approximately 2 cm (Figure 1B). This observation suggests that the action of the sgRNAs in the CRISPR/Cas9 library disrupted metastatic suppressor genes in the genome and that loss of function of these genes converted minimally metastatic cells to highly metastatic cells in different organs.

Next, we conducted next-generation sequencing on 111 samples, including 32 primary tumors, 15 recurrent tumors, and 16 metastatic tumors 8 weeks after implantation of 545-GFP-GeCKOv2 cells and 48 blood samples obtained from 2 weeks to 8 weeks after implantation. Compared with cells prior to implantation, which contained sgRNA for 14,806 genes, sgRNAs representing a total of 2593 genes were identified from 111 samples, including 1818 genes from metastatic tumors, and 162 genes shared among all types of samples (i.e., cells from primary tumors, recurrent tumors, blood samples, and metastatic tumors) (Supplemental Table 1; supplemental material available online with this article; https://doi.org/10.1172/JCI149473DS1) with a pattern of gradual reduction in the cells obtained from primary, recurrent tumors and 5 target organs (Figure 1C).

To define human homologous metastatic repressors in our mouse screening process, we first identified potential tumor suppressors in a TCGA-BRCA (The Cancer Genome Atlas Breast Invasive Carcinoma) data set that included data on 1100 patients with breast cancer and 112 healthy donors. We found 1521 genes that showed not only low expression compared with their expression in mammary tissues from healthy donors (*P* < 0.05) but also were linked to poor survival outcomes compared with the same genes with relatively high expression (*P* < 0.05) (Supplemental Figure 1B and Supplemental Table 2). After comparing the 1521 human genes with the 2593 candidate genes identified across all samples, we found 160 human homologous genes (Supplemental Figure 1C). The top 50 genes that might function in driving both tumorigenesis and metastasis are summarized according to the frequency of sgRNA appearance in reads (Supplemental Figure 1D). We also compared 1521 human genes with the 1818 genes identified from metastatic tumors in our screening and identified 117 human homologous genes that might be involved in metastatic processes (Figure 1D and Supplemental Table 3). The top 50 potential metastatic genes were ranked based on the frequency of their sgRNA appearance reads in the recurrent cancer, blood, and metastatic organs as determined by OncoPrint (https://github.com/jokergoo/ComplexHeatmap) (Figure 1E). Notably, from the analysis performed through 2 different approaches, we noticed that *ATP11b* was ranked at the top (Figure 1E and Supplemental Figure 1D). The analysis of the genome-wide screen showed that some genes appeared more frequently among different samples, and other genes exhibited organ-specific patterns; these differential distribution patterns may reflect the different roles of these genes in cancer metastasis.

### ATP11b is a potent metastatic suppressor associated with Brca1 deficiency.

**Figure 2:**
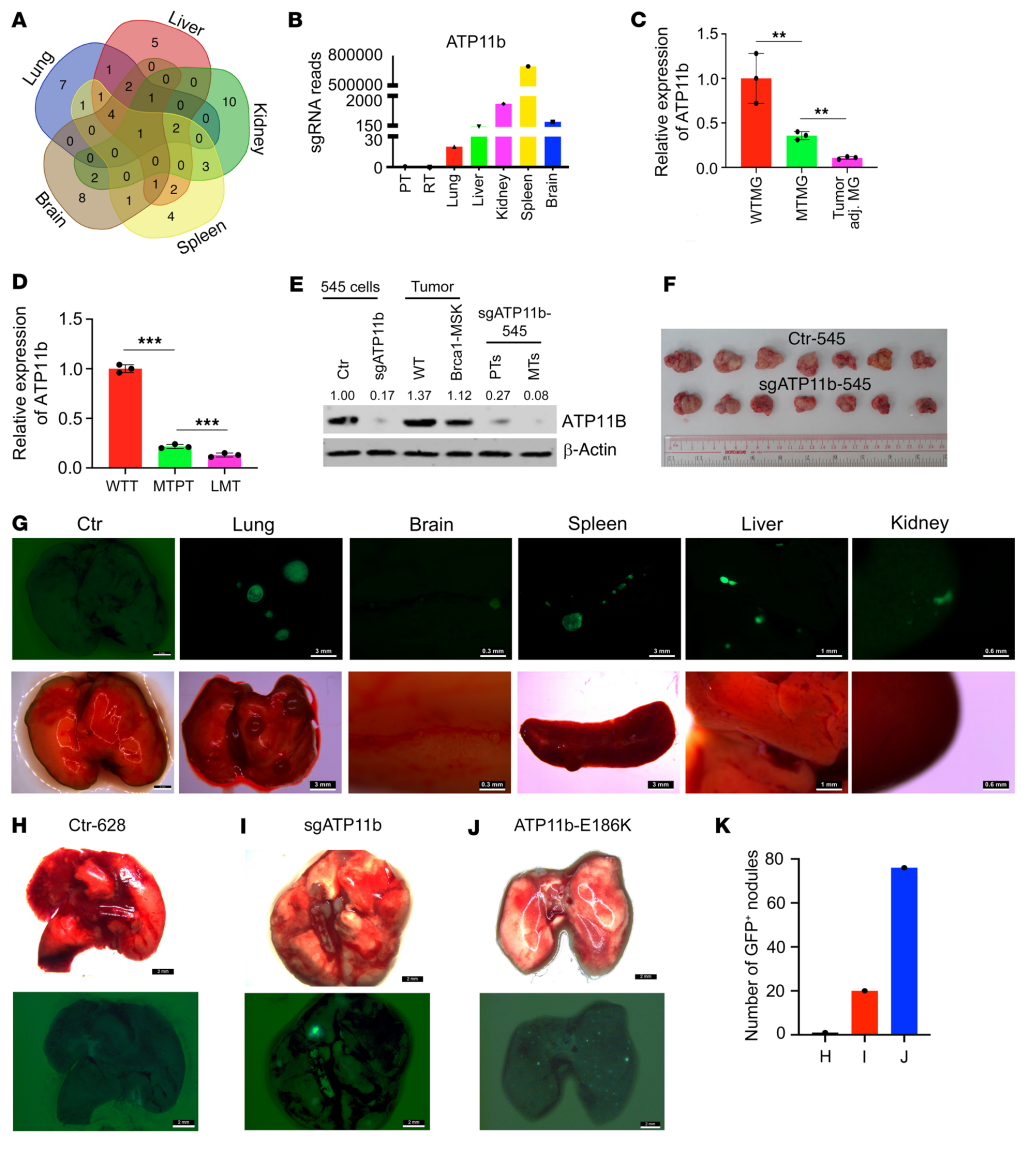
Metastasis of 545 cell tumors with sg*ATP11b* expression in multiple organs. (**A**) Venn diagram analysis of common genes in 5 metastatic organs from 117 genes in Figure 1D. (**B**) Reads of sg*AT11b* from 5 different metastatic organs compared with primary tumor. (**C**) Expression of *ATP11b* in WT mammary gland (WTMG), *Brca1*-MT mammary gland (MTMG), and tumor-adjacent mammary gland (*n* = 3 mice per group). (**D**) Expression of *ATP11b* in WT tumors (WTT), primary tumors from *Brca1*-MSK mice (MTPT), and lung metastatic tissues (LMT) from *Brca1*-MSK mice (*n* = 3 mice per group). (**E**) ATP11B protein levels from 545 cells without or with expression of sg*ATP11b* before injection (first two lanes), tumors from *Brca1*-WT mammary tumors (WT) and *Brca1*-MSK mice (third and fourth lanes, *n* = 3 pairs of mice), and primary breast tumors (PTs) and metastatic tumors (MTs) from sg*ATP11b*-545 nude mice (fifth and sixth lanes, *n* = 3 pairs of mice). (**F**) Images of primary tumors with or without sg*ATP11b* expression (*n* = 7 mice per group). (**G**) Representative images of lungs 8 weeks after fat pad implantation with parental 545 cells (Ctr) (*n* = 10 mice) (scale bar: 2 mm). Representative GFP-metastatic signals in lungs (*n* = 13 mice) (scale bar: 3 mm), brain (*n* = 2 mice) (scale bar: 0.3 mm), spleen (*n* = 3 mice) (scale bar: 3 mm), liver (*n* = 4 mice) (scale bar: 1 mm), and kidney (*n* = 3 mice) (scale bar: 0.6 mm) from 545 cells expressing sg*ATP11b* 8 weeks after fat pad implantation. (**H**–**J**) Representative images of lungs in parental cell control group (Ctr-628) (**H**), sg*ATP11b*-628 cells (scale bar: 2 mm) (**I**), and *ATP11b*-E186K-628 cells (**J**). (**K**) Quantification of metastatic nodules in **H**–**J** (*n* = 8 mice per group). Recipient mice were killed at day 21 after implantation of cells. Statistical data in **C** and **D** were assessed using 1-way ANOVA with Bonferroni’s multiple-comparison test; data are presented as mean ± SEM. **P < 0.01, ***P < 0.001.

Considering the observation of different sgRNA distribution patterns, we compared the sgRNA reads enriched with 117 human homologous genes in 5 metastatic organs, including lung, liver, spleen, brain, and kidney (Supplemental Figure 2A). Our analysis revealed that *ATP11b*-sgRNA was the only sgRNA that was highly enriched in all 5 organs (Figure 2, A and B, and Supplemental Table 4), while some other genes appeared in 2 to 3 organs (Figure 2A) or only 1 organ (Supplemental Table 4).

*ATP11b* is a flippase that is critical for the asymmetrical distribution of PS on the inner leaflet of the cell membrane (25, 26). Loss of *ATP11b* function allows PS to flip to the outer leaflet of the cell membrane to generate a global immunosuppressive signal (23). To verify the expression of *ATP11b* in mammary tissues of wild-type (WT) and *Brca1*-MSK mice, RNA from the WT mouse mammary gland, MT mammary gland, MT tumor-adjacent mammary gland, and tumor tissues from WT tumors, MT primary tumors, and MT metastatic tumors was extracted and sequenced. The data showed that the expression level of *ATP11b* in MT mammary glands was only 25% that in WT mammary glands and was even lower in tumor-adjacent mammary tissues (Figure 2C). *ATP11b* expression in *Brca1*-MT primary tumors was only 10% that in WT tumors as revealed by quantitative PCR (qPCR) and was much lower in lung metastatic tumor tissues (Figure 2D). Consistent with RNA expression, the protein level of ATP11B was lower in tumors from *Brca1*-MSK mice compared with its level in tumors from WT and *Trp53^+/Co^ MMTV-Cre* (*Trp53*-MSK) mice (Figure 2E and Supplemental Figure 2B), and it was further reduced in sg*ATP11b*-545 cells and metastatic tumors from sg*ATP11b*-545–carrying mice compared with their respective controls (Figure 2E).

To validate the function of *ATP11b* in suppressing tumor metastasis in vivo, we implanted parental 545 cells and sg*ATP11b*-545 cells into the mammary fat pad of nude mice. Notably, the metastatic phenotypes were recapitulated in multiple organs 8 weeks after the implantation of sg*ATP11b*-545, but not parental 545 cells, although there was no obvious difference in the size of the primary tumors (Figure 2F). Among the mice with a metastatic phenotype, 13 had lung metastasis, 2 had brain metastasis, 3 had spleen metastasis, 3 had kidney metastasis, and 4 had liver metastasis (Figure 2G). Sanger sequencing confirmed targeted mutation of *ATP11b* (Supplemental Figure 2C). Consistently, analyzing the Gene Expression Omnibus (GEO) data set GSE61304 revealed that breast cancer patients with low levels of *ATP11B* had worse survival outcomes after diagnosis (Supplemental Figure 2D). These data demonstrate that *ATP11B* is a potent metastatic suppressor and that its disruption enhances tumor metastasis to many organs.

ATP11B contains 4 main domains: cytoplasmic A (actuator), N (nucleotide binding), P (phosphorylation), and R domains. A DGET motif (amino acids 178–181) in the A domain facilitates the dephosphorylation of the phosphorylated intermediate, which will affect the catalytic activity (25). Information obtained from the cBioPortal database indicated that a patient with a point mutation in the DGET motif, ATP11B-E180K, which may disrupt the catalytic activity of the protein, had lymph node metastasis. To investigate whether overexpression of the catalytically dead ATP11B could phenocopy ATP11B^lo^ or *ATP11B*-MT state in PS displacement and tumor metastasis, we generated an E186K mutation in mouse *ATP11b*, which is equivalent to human E180K in the catalytic domain of ATP11B (Supplemental Figure 2E), and compared the effect of sg*ATP11b* and *ATP11b*-E186K in 545 cells. The FACS analysis with PS antibody showed that the transfection of sg*ATP11b* or *ATP11b*-E186K achieved a comparable increase of PS displacement on the outer leaflet of the cell membrane in the transfected cells (Supplemental Figure 2, F and G). A similar finding was observed in another *Brca1*-MT mammary tumor cell line, 628, which was derived from a *Brca1*-MSK mouse with multiorgan metastasis. This line had a higher level of PS than 545 and could form metastatic foci in the lung after 4 weeks of fat pat implantation. The data also revealed a comparable increase of PS in the 628 cells transfected with sg*ATP11b* or *ATP11b*-E186K (Supplemental Figure 2, H and I). Next, we implanted Ctr-628, sg*ATP11b*-628, and *ATP11b*-E186K-628 cells into the fat pad of nude mice (*n* = 8 per group) and sacrificed the recipient mice 3 weeks after the implantation, when the Ctr-628 generated very few or no visible metastatic nodules in the lung. In contrast, both sg*ATP11b*-628 and *ATP11b*-E186K-628 significantly enhanced lung metastasis (Figure 2, H–K). These data suggest that the impaired catalytic function of ATP11B is responsible for the enhanced PS displacement and tumor metastasis.

### Low levels of ATP11b expression induce nonapoptotic PS in the outer leaflet of the cell membrane.

**Figure 3:**
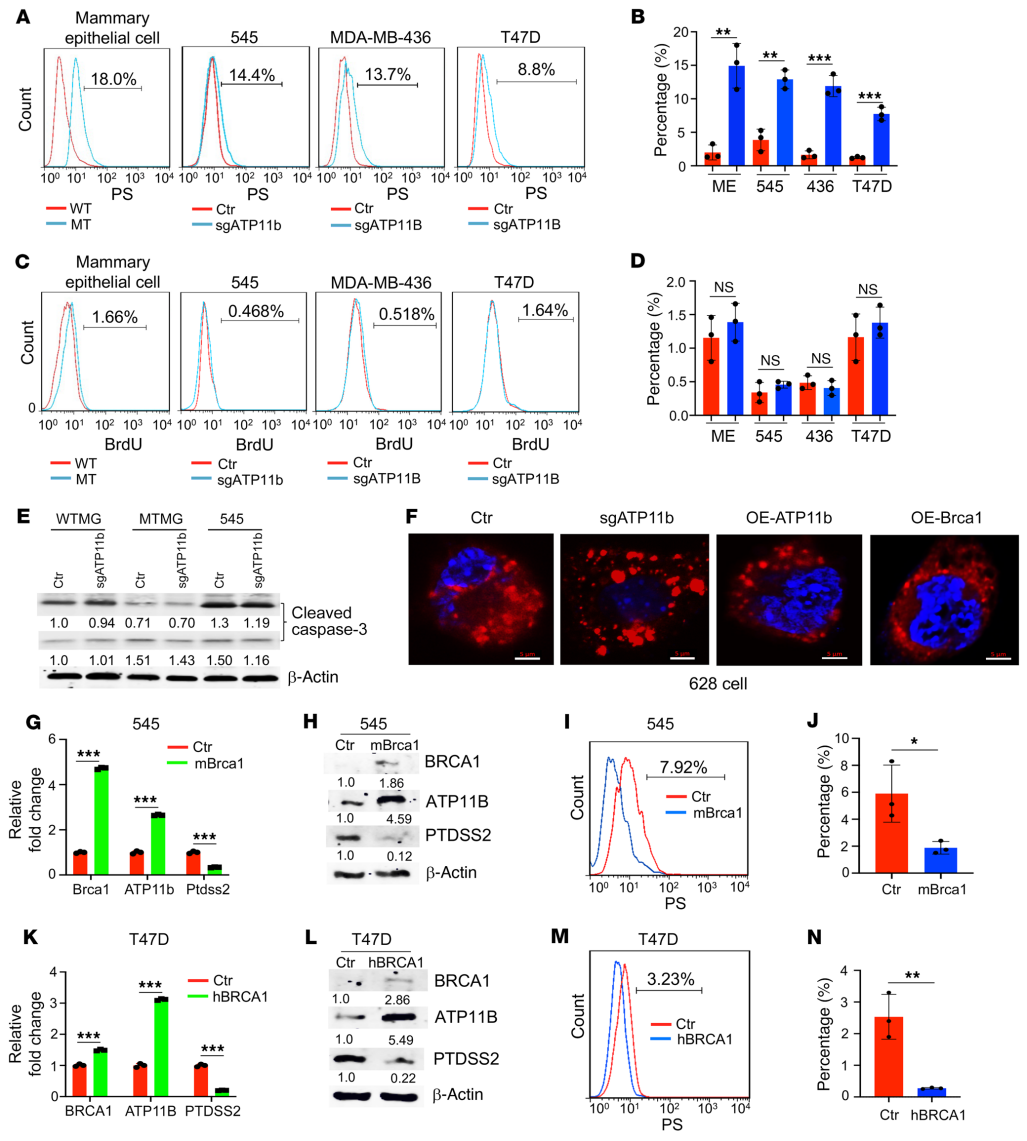
PS displacement on outer leaflet of the cell membrane is regulated by ATP11B and BRCA1. (**A**) PS displacement on the cell membrane by FACS with anti-PS antibody in WT and *Brca1*-MT primary mammary epithelial cells (*n* = 4 pairs) and 545, MDA-MB-436, and T47D cells without or with sg*ATP11B* expression. (**B**) Quantification for **A**. (**C**) Apoptotic cells in WT and *Brca1*-MT primary mammary epithelial cells (*n* = 4 pairs) and 545, MDA-MB-436, and T47D cells without or with sg*ATP11B* expression by FACS using the APO-BrdU Kit. (**D**) Quantification for **C**. (**E**) Protein levels of cleaved caspase-3 in WT (B477) and *Brca1*-MT (G600) mammary epithelial cells and 545 *Brca1*-MT tumor cells without or with sg*ATP11b* expression. (**F**) Representative images of PS displacement in 628 parental cells and 628 cells with sg*ATP11b* expression, overexpression (OE) of *ATP11b*, and OE-*Brca1* stained with PS antibody and DAPI, imaged by Zeiss LSM 880 high-resolution microscope with Airyscan (*n* = 3 times). Scale bars: 5 μm. (**G** and **H**) *Brca1*, *ATP11b*, and *Ptdss2* expression in 545 cells without (red) or with m*Brca1* cDNA expression (green) by qPCR (**G**) and protein levels by Western blot (*n* = 3 times) (**H**). (**I** and **J**) PS displacement on cell membrane of 545 cells without (red) or with (blue) m*Brca1* cDNA expression by FACS analysis (**I**), and quantification (**J**) (*n* = 3 times). (**K** and **L**) h*BRCA1*, *ATP11B*, and *PTDSS2* expression in T47D cells without (red) or with h*BRCA1* cDNA expression (green) by qPCR (**K**), and protein levels by Western blot (*n* = 3 times) (**L**). (**M** and **N**) PS displacement on cell membrane of T47D cells without or with h*BRCA1* cDNA expression by FACS (**M**), and quantification (**N**) (*n* = 3 times). Error bars show mean ± SEM of 2-tailed Student’s *t* test. *P < 0.05, **P < 0.01, ***P < 0.001.

Next, we investigated the mechanism by which the disruption of *ATP11b* enhances tumor metastasis. As the main function of *ATP11b* is to maintain PS in the cytoplasmic side at the expense of ATP through its flippase activity (25, 26), we reasoned that the markedly reduced level of ATP11B or loss of ATP11B would increase the abundance of PS on the outer leaflet of the cell membrane. To test this hypothesis, the PS signals on the cell membrane were investigated by FACS analysis from primary mammary epithelial cells in both WT and *Brca1*-MSK mice, using multiple cell lines without or with the expression of sg*ATP11b*, including mouse *Brca1*-MT (G600) mammary epithelial cells, 545 *Brca1*-MT tumor cells, and 628 *Brca1*-MT tumor cells, as well as MDA-MB-436 and T47D *BRCA1*-WT cells. The data revealed more PS on the outer leaflet of the cell membrane of mammary epithelial cells in *Brca1*-MSK mice compared with WT mice and in cell lines expressing sg*ATP11b* (Figure 3, A and B, and Supplemental Figure 3, I and J) with 2 or 3 different sgRNAs (Supplemental Figure 3, A–F) compared with their respective controls.

PS can be flipped to the outer leaflet of the plasma membrane by scramblase XKR8, which is activated by caspase cleavage in apoptotic cells (27). Indeed, FACS analysis using an antibody against annexin V confirmed increased PS in the outer member of these cells (Supplemental Figure 3, G–J) without an increase in apoptotic cell populations with an APO-BrdU Kit (Novus Biologicals company, catalog NBP2-31161) in any of the 5 cell lines (Figure 3, C and D, and Supplemental Figure 3, I and J). Consistently, protein levels of cleaved caspase-3 were not increased in the 3 different cell lines expressing sg*ATP11b* (Figure 3E).

We next investigated whether levels of PS are affected by *ATP11b* or *Brca1*. We first stained mammary glands in 10-month-old mice with anti-PS antibody and detected much stronger PS staining in *Brca1*-MSK mice than in WT mice (Supplemental Figure 3K). To determine whether PS flipping to the outer leaflet of the cell membrane is affected by the expression of sg*ATP11b* and *Brca1*, we examined PS displacement in *Brca1*-MT cells expressing sg*ATP11b* or overexpressing (OE) *ATP11b* or *Brca1* by using immunofluorescence staining and imaging by high-resolution microscope. The data revealed that PS on the outer leaflet of the cell membrane was increased in *Brca1*-MT cells expressing sg*ATP11b* compared with control cells and was decreased in cells with either OE-*ATP11b* or OE-m*Brca1* (Figure 3F). To determine whether the level of PS on the outer leaflet of the cell membrane could be suppressed by *BRCA1* expression, we introduced *BRCA1* cDNA into 545, T47D, and MDA-MB-436 cells and examined the PS levels on their membranes. The data revealed that overexpression of BRCA1 increased ATP11B expression and protein level (Figure 3, G, H, K, and L, and Supplemental Figure 3, L and M) and reduced PS levels in all 3 cell lines (Figure 3, I, J, M, and N, and Supplemental Figure 3, N and O), suggesting that the BRCA1 protein either contributes to the maintenance of the ATP11B protein or inhibits the expression of genes that are critical for PS synthesis in both *BRCA1*-WT and *BRCA1*-MT cells. All these data demonstrate that low levels of *ATP11b* expression induce an increase in nonapoptotic PS on the outer leaflet of the cell membrane and the PS population on the outer leaflet of the cell membrane is reduced upon *Brca1* expression.

### Elevated PTDSS2 continuously provides PS in Brca1-MT cells.

**Figure 4:**
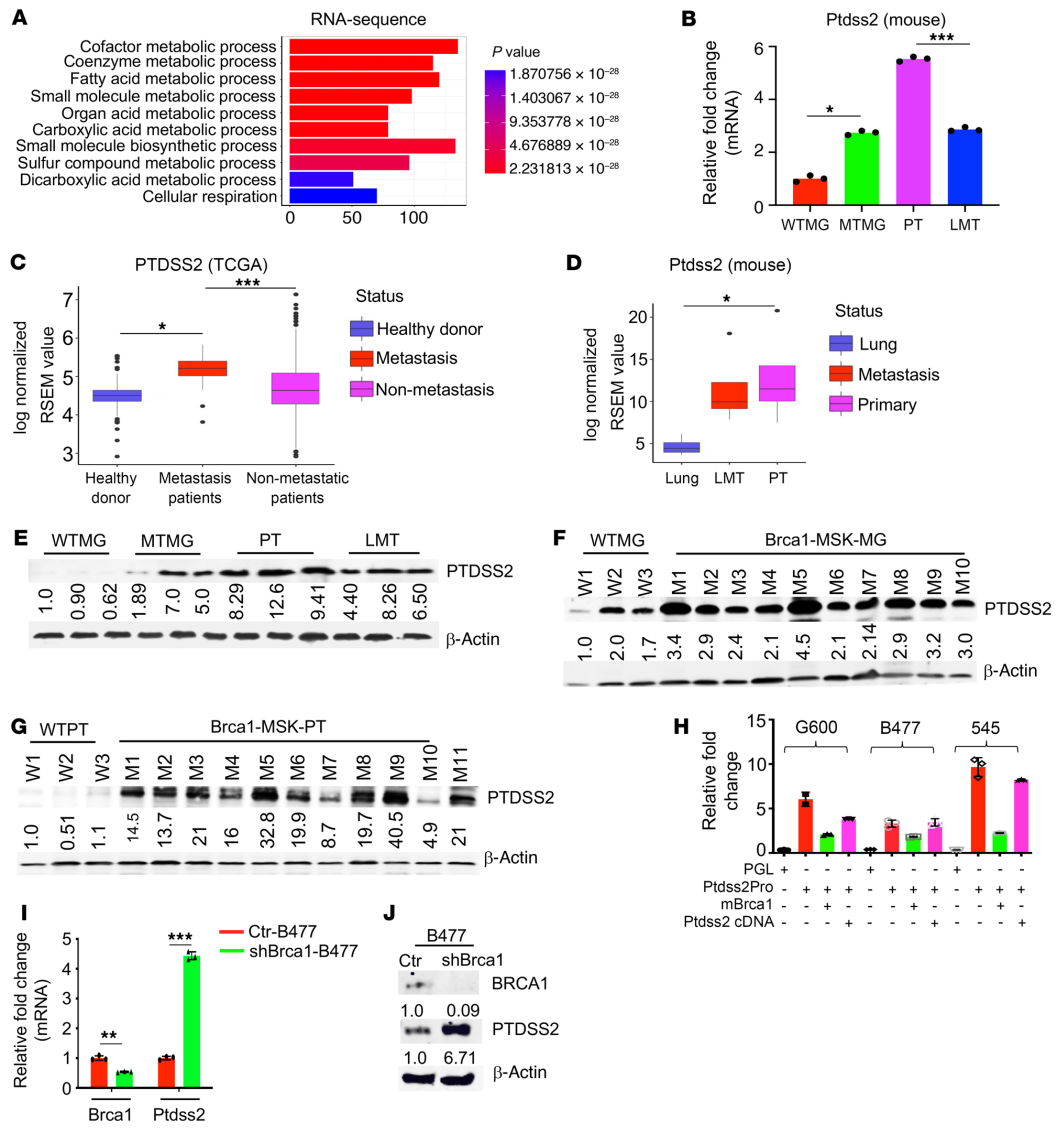
*Ptdss2* is negatively regulated by *Brca1*. (**A**) The top 10 biological processes in *Brca1*-MT mammary tissues annotated by GO analysis with comparison of RNA-Seq from MTMG, PT, and LMT of *Brca1*-MSK mice versus WTMG (*n* = 3 mice per group). (**B**) *Ptdss2* expression in WT lung and metastatic and primary tumors (*n* = 3 mice per group). (**C**) Expression of *PTDSS2* in human breast cancer patients with or without metastasis compared with healthy donors in TCGA-BRCA database (healthy donors, *n* = 122; non-metastatic patients, *n* = 623; metastatic patients, *n* = 9). (**D**) *Ptdss2* expression from bulk RNA-Seq in lung, LMT, and PT compared with WT controls (*n* = 3 mice per group). RSEM, RNA-Seq by Expectation–Maximization. (**E**) Protein levels of PTDSS2 in WTMG, MTMG, PT, and LMT by Western blot (*n* = 3 times). (**F** and **G**) Protein levels of PTDSS2 in both mammary gland (**F**) and tumors (**G**) with age-matched WTMG (*n* = 3 mice) and MTMG (*n* = 10 mice), WT tumor (*n* = 3 mice), and PT from *Brca1*-MSK mice (*n* = 11 mice) by Western blot (*n* = 3 times). (**H**) *Ptdss2* promoter activity assay in G600, B477, and 545 cells without or with expression of m*Brca1* cDNA or m*Ptdss2* cDNA (*n* = 3 times). (**I** and **J**) Expression of *Brca1* and *Ptdss2* by qPCR (**I**) and Western blot (**J**) in B477 cells without (red) or with (green) sh*Brca1* cDNA (*n* = 3 times). Statistical data in **B**–**D** were assessed using 1-way ANOVA with Bonferroni’s multiple-comparison test; data are presented as mean ± SEM. *P < 0.05, **P < 0.01, ***P < 0.001.

To explore the source of PS that supports its constitutive exposure in *Brca1*-MT mammary epithelial cells, we hypothesized that PS synthesis might be abnormally upregulated in *Brca1*-MT cells, since *Brca1* is a transcription factor. Our Gene Ontology (GO) analysis of the enrichment of genes in biological process with RNA expression files from mammary glands and primary and metastatic tumors in *Brca1*-MSK and WT mice revealed that fatty acid metabolic processes were highly activated (Figure 4A and Supplemental Table 5). PS contains 2 long fatty acid chains that are attached to the first and second carbons of glycerol and a serine attached to the third carbon of glycerol through a phosphodiester linkage (28, 29). PS is produced from phosphatidylcholine via phosphatidylserine synthase 1 (*Ptdss1*) and from phosphatidylethanolamine via phosphatidylserine synthase 2 (*Ptdss2*), where *Ptdss1* and *Ptdss2* are catalytic enzymes of PS synthesis (30). Thus, we examined the expression of *Ptdss1* and *Ptdss2*, as well as other major enzymes in the PS synthesis pathway, including acetyl-CoA carboxylase-α (*Acaca*) and fatty acid synthase (*Fasn*), by qPCR and Western blot analysis to determine whether their expression and protein levels were affected by *Brca1* deficiency. We found that the expression of *Acaca*, *Ptdss1*, and *Ptdss2*, but not *Fasn*, was increased not only in *Brca1*-MT mammary glands but also in both primary tumors and lung metastatic tissues of *Brca1*-MSK mice compared with WT mice (Figure 4B and Supplemental Figure 4, A–C). The analysis of gene expression files from TCGA data set and mouse data showed that expression of *PSS2* is increased in breast cancer patients and mice with metastasis compared with that in their controls (Figure 4, C and D). Western blot analysis showed that only the PTDSS2 protein level was stably elevated from both *Brca1*-MT mammary glands (Figure 4, E and F) and tumor tissues of *Brca1*-MSK mice (Figure 4G), but not PTDSS1 (Supplemental Figure 4D). Therefore, we decided to further examine whether *Brca1* can regulate *Ptdss2* at the transcription level.

To investigate this possibility, the *Ptdss2* promoter was cloned into a PGL3 luciferase reporter, and promoter activity was examined under different conditions in 2 *Brca1*-MT cell lines (545 and G600 cells) and a control cell line. We found that the *Ptdss2* promoter had higher activity in *Brca1*-MT 545 and G600 cells than in WT cells; this activity could be significantly suppressed when m*Brca1* cDNA was overexpressed in these cells, and it was restored more in the 2 *Brca1*-MT cell lines than in the WT cells when the *Ptdss2* cDNA construct was introduced (Figure 4H), suggesting that *Ptdss2* promoter activity can be strongly suppressed by *Brca1*. To further support this observation, we knocked down *Brca1* in WT cells and overexpressed m*Brca1* in *Brca1*-MT cells. We detected increased mRNA expression and protein levels of PTDSS2 in the sh*Brca1*-WT cells (Figure 4, I and J) and reduced expression and protein levels of PTDSS2 in the *Brca1*-MT cells with overexpression of m*Brca1* cDNA (Figure 3, G and H) and in the T47D and MDA-MB-436 cells with expression of h*BRCA1* (Figure 3, K and L, and Supplemental Figure 3, L and M).

**Figure 5:**
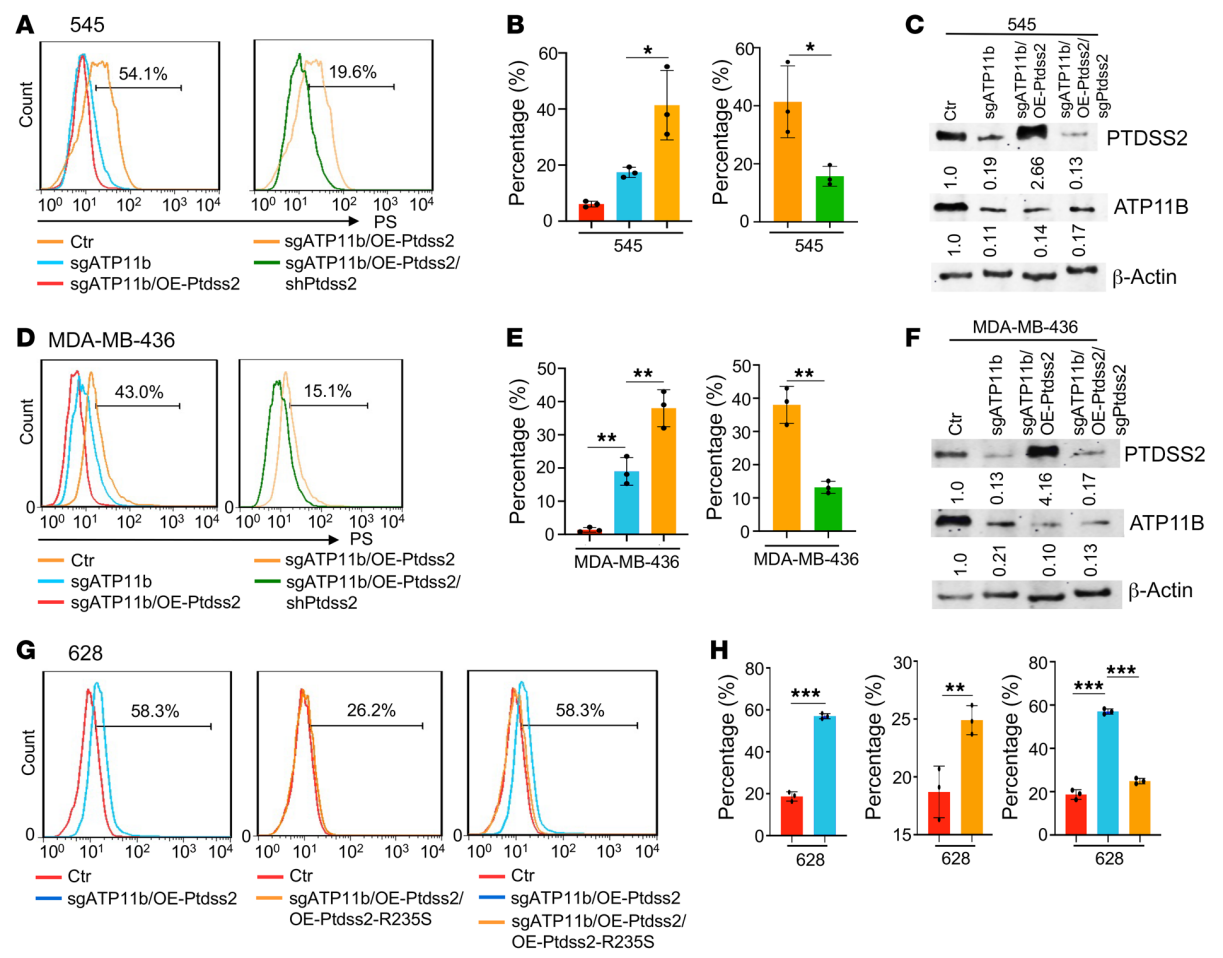
Overexpression of *Ptdss2* and sg*ATP11b* increases PS on the cell membrane. (**A**–**F**) PS displacement on cell membrane of 545 (**A**–**C**) and MDA-MB-436 (**D**–**F**) cells without (red) or with sg*ATP11b* (blue), sg*ATP11b*/OE-*Ptdss2* (orange), or OE-sh*Ptdss2* in sg*ATP11b*/OE-*Ptdss2* cells (dark green) by FACS with anti-PS antibody (**A**), quantification (**B** for part **A** and **E** for part **D**), and protein levels of PTDSS2 and ATP11B in the above cells (**C** and **F**) (*n* = 3 times). (**G** and **H**) PS displacement in sg*ATP11b*/OE-*Ptdss2-*628 and sg*ATP11b*/OE-*Ptdss2*/OE-*Ptdss2*-R235S-628 cells and overlap of the 2 plots, and quantification (**H**) (*n* = 3 times). Statistical data in the left side of **B **and** E** and the right side of **H** were analyzed by 1-way ANOVA with Bonferroni’s multiple-comparison test; the rest of the statistical data were analyzed by 2-tailed Student’s *t* test; data are presented as mean ± SEM. *P < 0.05, **P < 0.01, ***P < 0.001.

To further investigate whether the PS level is affected by *Ptdss2* on the cell membrane upon loss of *ATP11b*, we first overexpressed *Ptdss2* cDNA (OE-*Ptdss2*) in 545 and MDA-MB-436 cells that had previously expressed sg*ATP11b*, and then we expressed sh*Ptdss2* in the cells expressing sg*ATP11b*/OE-*Ptdss2* (Supplemental Figure 4, E–H). We found that the PS populations were increased in all 3 cell lines upon the expression of sg*ATP11b*, and the PS populations were further increased when *Ptdss2* was overexpressed in these cells (Figure 5, A–F, and Supplemental Figure 4, I–L), as determined by FACS analysis with both anti-PS and anti–annexin V antibodies. The PS populations were also reduced when we expressed sh*Ptdss2* or sh*PTDSS2* in 545, MDA-MB-436, and G600 cell lines together with sg*ATP11B*/OE-*PTDSS2*, as revealed by FACS analysis with anti-PS and anti–annexin V antibodies (Figure 5, A–F, and Supplemental Figure 4, I–L). The same observations were confirmed in the *BRCA1*-WT T47D cells subjected to anti-PS antibody staining (Supplemental Figure 4, M and N). PS populations were suppressed on the outer membranes of the 545, MDA-MB-436, and T47D cells when *BRCA1* cDNA was overexpressed (Figure 3, I, J, M, and N, and Supplemental Figure 3, N and O). Next, we investigated whether ATP11B^lo^PTDSS2^hi^ state could be reversed by overexpression of catalytically dead PTDSS2. Using IUPred2A (31) software, we found that the PSS domain in PTDSS2 may be a potential catalytic domain. We then tested the function of *PTDSS2*-R235S in mice, which corresponds to human PTDSS2-R313C (Supplemental Figure 4O). The data revealed that OE-*Ptdss2*-R235S could override increased PS displacement on the cell membrane induced by the expression of sg*ATP11b*/OE-*Ptdss2* in 628 (Figure 5, G and H), 545 (Supplemental Figure 4, P and Q), and EMT6 (Supplemental Figure 4, R and S) cells.

In summary, these data demonstrate that *Brca1* serves as a suppressor of *Ptdss2* and an elevated *Ptdss2* level in *Brca1*-deficient cells contributes to the constitutively available PS in *Brca1*-MT cells.

### ATP11B^lo^ and PTDSS2^hi^ enhance breast cancer metastasis.

**Figure 6:**
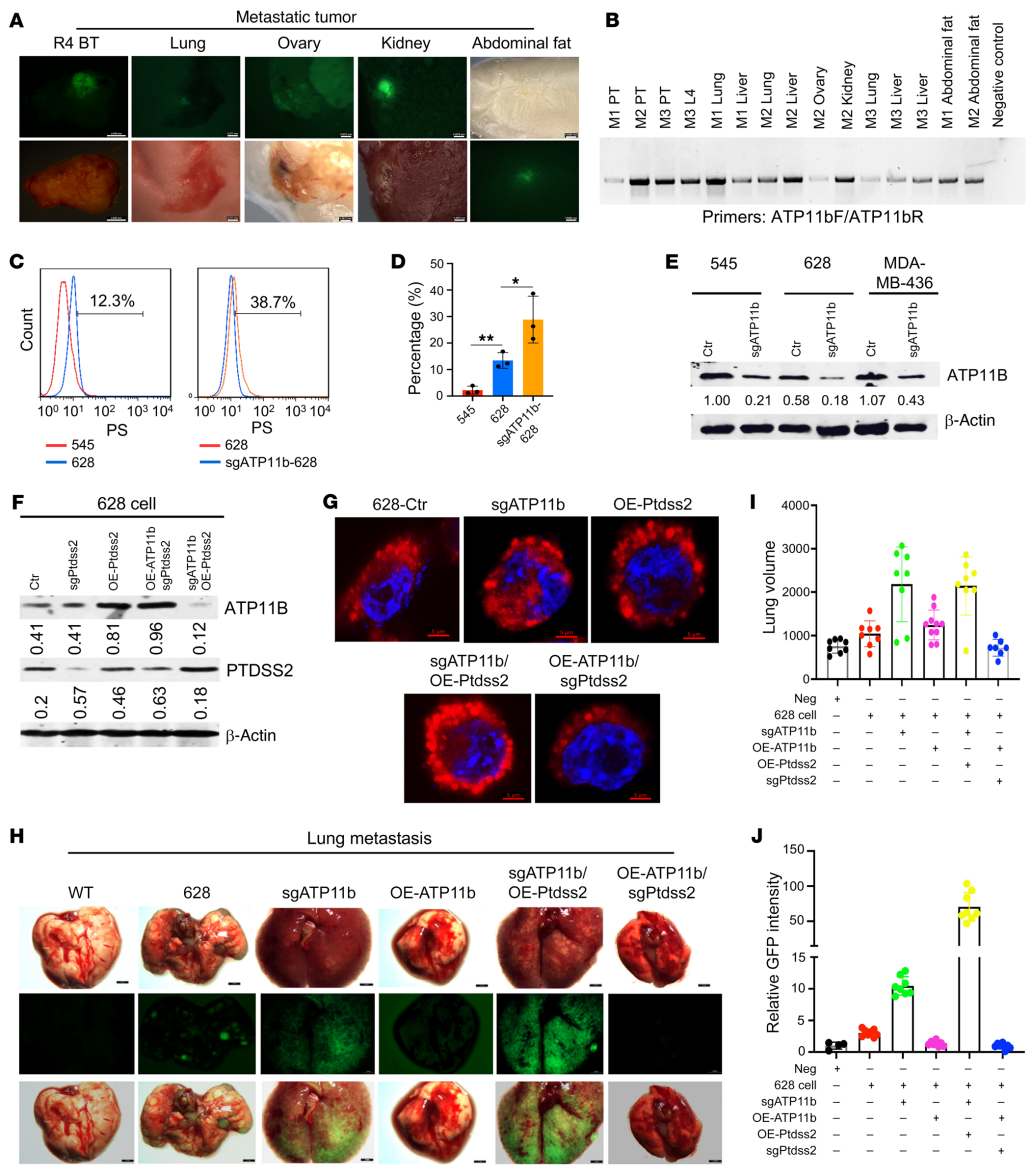
ATP11b^lo^PTDSS2^hi^ expression enhances breast cancer metastasis. (**A**) Representative metastatic images of primary breast tumor, lung, ovary, kidney, and abdominal fat from a 7-month-old *Brca1*-*Trp53*-MSK female mouse after intraductal injection of mixed sg*ATP11b* and *Ptdss2*-GFP lentiviruses (*n* = 5 mice). Scale bars: 2.548 mm (R4 BT), 0.277 mm (lung), 0.3612 mm (ovary), 0.4676 mm (kidney), 0.9263 mm (abdominal fat). (**B**) Validation of sg*ATP11b* DNA in primary tumor tissues and metastatic tissues, including lung, liver, ovary, kidney, and abdominal fat tissues, as determined with specific primers for *ATP11b* by PCR. (**C** and **D**) PS displacement in 545 and 628 cells without or with sg*ATP11b* expression by FACS analysis with PS antibody. (**D**) Quantification of PS on the outer cell membrane in **C** (*n* = 3). (**E**) Protein levels of ATP11B in 545, 628, and MDA-MB-436 cells without or with sg*ATP11b* expression by Western blot (*n* = 3). (**F**) Protein levels of ATP11B and PTDSS2 in 545 and 628 cells without or with sg*Ptdss2*, OE-*ATP11b*, OE-*ATP11b*/sg*Ptdss2*, or sg*ATP11b*/OE-*Ptdss2* expression by Western blot (*n* = 3). (**G**) Representative images of PS displacement in/on the membranes of 628 cells without or with sg*ATP11b*, OE-*Ptdss2*, sg*ATP11b*/OE-*Ptdss2*, or OE-*ATP11b*/sg*Ptdss2* expression by immunofluorescence staining with an anti-PS antibody (red), imaged by super-resolution microscopy (*n* = 3). Scale bars: 5 μm. (**H**) Representative lung bright-field (BF) images, GFP signal, and overlapped images showing the BF and GFP signals in nude mice without implantation (WT) and 3 weeks after mammary fat pad implantation with 628 parental cells or 628 cells expressing sg*AT11b*, or with OE-*ATP11b* in sg*ATP11b* cells, or sg*ATP11b*/OE-*Ptdss2*, or OE-*ATP11b*/sg*Ptdss2* (*n* = 3). Scale bars: 3 mm. (**I** and **J**) Quantification for lung volumes (**I**) and GFP intensities (**J**) from the same cohort of mice shown in **H** (*n* = 8 mice per group). Statistical data in **D** were analyzed by 1-way ANOVA with Bonferroni’s multiple-comparison test; data are presented as mean ± SEM. *P < 0.05, **P < 0.01.

To explore whether OE-*Ptdss2* and sg*ATP11b* have a cooperative effect on tumor metastasis in *Brca1*-*Trp53*-MSK mice, we first performed intraductal injection of virus expressing sg*ATP11b*, mixed viruses expressing either sg*ATP11b* or *Ptdss2*-GFP, or control lenti-v2 virus into 2-month-old *Brca1^Co/Co^ p53^+/Co^ MMTV-Cre* (*Brca1*-MSK *Trp53*-MSK) mice. Since approximately 20% of these mice develop mammary tumors at approximately 9–12 months of age (Supplemental Figure 1A), which serves as a reference for endogenous tumor metastasis (6, 8, 12, 32, 35, 37, 41), we examined tumor metastasis 5 months after intraductal infection with lentiviruses with different genotypes. The experimental data revealed that GFP metastatic signals were detected in multiple organs, including the lung, liver, abdominal fat, kidney, ovary, and mammary tissues, in all 5 primary tumor–bearing mice injected with mixed viruses, whereas only 1 mouse with lung metastasis was observed among 4 primary tumor–bearing mice expressing only sg*ATP11b* (Figure 6, A and B, and Supplemental Figure 5A), and no metastasis was observed in the control lenti-v2–injected mice. Deletions within the *ATP11b* gene were confirmed by Sanger sequencing of samples obtained from 3 different metastatic organs (Supplemental Figure 5B), and mutations in *ATP11b* (Figure 6B) or deletions in *Brca1*-MT cells (Supplemental Figure 5C) were detected in both primary tumors and metastatic lungs in mice injected with a mixture of viruses, demonstrating that loss of function of *ATP11b* together with OE-*Ptdss2* in mammary epithelial cells dramatically enhances metastasis.

To further investigate the effects of *ATP11b* and *Ptdss2* on tumor metastasis, we used 628-GFP cells, in which a metastatic GFP signal could be detected in the lung 4 weeks after orthotopic injection because 628 cells have a significantly higher PS population on the cell membrane than 545 cells, as revealed by FACS analysis (Figure 6, C and D), and lower ATP11B protein levels than 545 cells, as determined by Western blotting (Figure 6, E and F). The levels of the PS population on the cell membrane were further increased in 628 cells expressing sg*ATP11b* (Figure 6, C and D).

Next, we monitored the PS location on the cell membrane (Figure 6G) and metastatic signals in nude mice (Figure 6H) as detected in 628-GFP cells with different combinations of *ATP11b* and *Ptdss2* expression, including 628-GFP-Ctr, sg*ATP11b*, OE-*Ptdss2*, OE-*ATP11b*, sg*ATP11b*/OE-*Ptdss2*, and OE-*ATP11b*/sg*Ptdss2*, both in vitro and in vivo. The PS population on the outer cell membrane was increased in 628 cells with sg*ATP11b* or OE-*Ptdss2* expression compared with the 628-GFP control cells, and the increase was much more in cells with both sg*ATP11b* and OE-*Ptdss2* expression (Figure 6G). In contrast, the PS displacement to the outer membrane was greatly reduced in 628-GFP cells with OE-*ATP11b*/sg*Ptdss2* (Figure 6G). GFP metastatic signals in the lung were detected by measurement of 628-GFP, and for every group with sg*ATP11b* expression, the GFP intensity was increased, but it was greatly reduced in the group of OE-*ATP11b* cells 3 weeks after implantation in the mammary fat pad (Figure 6H). Notably, the lung volume in the sg*ATP11b*/OE-*Ptdss2* and sg*ATP11b* groups was increased significantly with strong GFP signals in comparison with other groups (Figure 6, I and J), even though there were no obvious differences in the growth of primary tumors in all groups (Supplemental Figure 5, D–I). In contrast, the metastatic signals and lung volumes were minimized in the groups with OE-*ATP11b* compared with the groups expressing either sg*ATP11b* or sg*ATP11b*/OE-*Ptdss2* (Supplemental Figure 5, E–I). The same effect of ATP11B on metastasis was also observed in EMT6 cells expressing sg*ATP11b* (Supplemental Figure 5, J–L), demonstrating that increased PS signal on the outer leaflet can also enhance metastasis in *Brca1*-WT mice.

Next, we investigated whether metastatic phenotype could be reversed by overexpression of catalytically dead *Ptdss2*-R235S mutation. The results showed that OE-*Ptdss2*-R235S completely suppressed the lung metastasis induced by sg*ATP11b*/OE-*Ptdss2*-628 cells (Supplemental Figure 5, M–Q). These data demonstrate that constitutive exposure of nonapoptotic PS on the outer leaflet of the cell membrane also requires the catalytic function of PTDSS2, which enhances breast cancer metastasis.

### Nonapoptotic PS signaling enhances the TME, benefiting tumor growth and metastasis.

**Figure 7:**
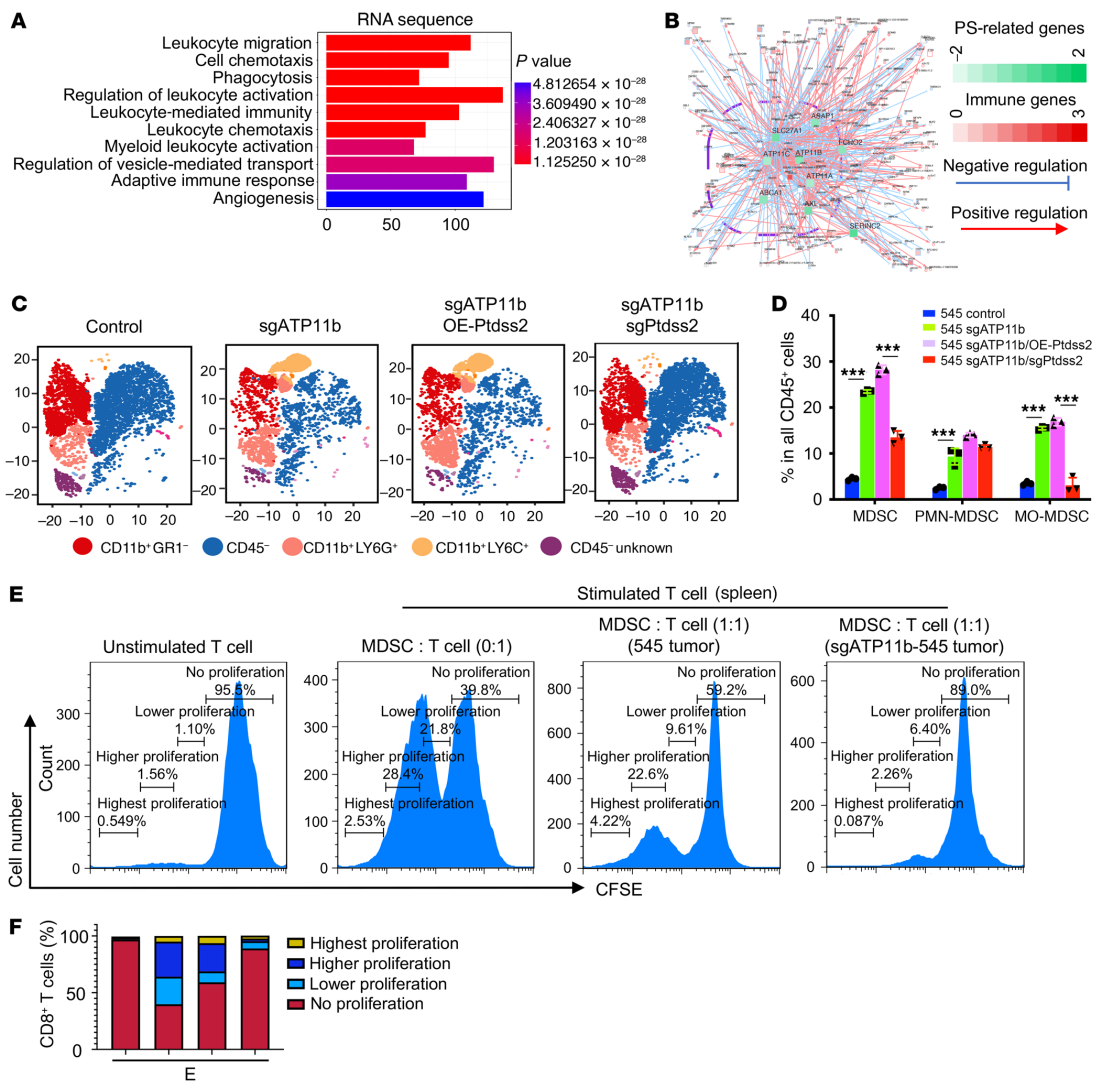
TME in mammary tissues with expression of sg*ATP11b* and sg*ATP11b*/OE-*Ptdss2*. (**A**) Top 10 activated pathways based on gene expression profiles of RNA-Seq from metastatic lungs compared with those of normal lungs (*n* = 4 mice per group) by the GO enrichment analysis in the clusterProfiler package (https://bioconductor.org/packages/release/bioc/html/clusterProfiler.html). (**B**) Master regulatory gene analysis of the immune gene transcriptional network established by the RTN package. The immune genes (red) and master regulatory genes (green) were extracted from the GSE54219 data set and analyzed by master regulatory analysis (MRA) (76 patients in BRCA1-WT group and 47 patients in BRCA1-MT group). Genes that are positively (red line) or negatively (blue line) regulated by PS-related genes are indicated. The *P* value of *ATP11B* as the master regulatory gene of immune cells is 0.0021. (**C**) Profiles of MDSC populations from mammary tumors implanted with 545 cells with indicated modifications in nude mice analyzed with antibodies against CD45, CD11B, Ly6G, and Ly6C by CyTOF analysis (*n* = 3 mice per group). (**D**) Quantification of MDSC, PMN-MDSC, and monocytic MDSC (MO-MDSC) populations in **C**. (**E** and **F**) Representative CFSE flow cytometry histograms showing inhibitive effect of MDSCs isolated from the spleen of tumor-bearing mice implanted with Ctr-545 or sg*ATP11b-*545 cells on the proliferation of T cells stimulated with anti-CD3/anti-CD28 antibody obtained from WT mice (**E**), and summarized results (**F**) (*n* = 3 mice per group). Statistical data in **D** were analyzed by 1-way ANOVA with Bonferroni’s multiple-comparison test; data are presented as mean ± SEM. ***P < 0.001.

To understand how the increased nonapoptotic PS signal enhances metastasis, we sought to determine whether the externalized nonapoptotic PS signal regulates the gene expression of immune cells. We first performed RNA sequencing analysis by comparing metastatic lungs from *Brca1*-MSK mice with lungs from WT mice using the Database for Annotation, Visualization and Integrated Discovery (DAVID) (Supplemental Table 5) and found that 1643 upregulated genes contributed to 7 upregulated innate immune responses and 1 adaptive immune response (Figure 7A). To further define master genes that are critical for the regulation of immune genes in breast cancer patients with *BRCA1* deficiency, we analyzed the GSE54219 data set with the GEOquery (https://www.bioconductor.org/packages/release/bioc/html/GEOquery.html) and RTN packages (https://www.bioconductor.org/packages/release/bioc/html/RTN.html) and found that *ATP11B* is one of the potent master genes related to PS function and could regulate immune gene expression in *BRCA1* mutation carriers (Figure 7B and Supplemental Table 6). These analyses suggest that increased PS on the outer leaflet of the cell membrane can affect immune cell function by either upregulating (red) or downregulating (blue) the expression of the genes in immune cells.

To explore whether the changed gene expression in *Brca1*-MT epithelial cells has an effect on the function of immune cells, we next performed cytometry by time of flight (CyTOF) analysis to examine myeloid-derived suppressor cells (MDSCs) from tumor tissues with Ctr-545 cells or 545 cells expressing sg*ATP11b*, sg*ATP11b*/OE-*Ptdss2*, or sg*ATP11b*/sg*Ptdss2* in nude mice using a combination of antibodies, including anti-CD45, anti-CD11b, anti-Ly6G, and anti-Ly6C. We detected a dramatic increase in both monocytic MDSC (CD11b^+^Ly6C^+^) and PMN-MDSC (CD11b^+^Ly6G^+^) subpopulations in the sg*ATP11b*-545 tumors compared with the 545 control tumors, and these populations were increased further in tumors expressing sg*ATP11b*/OE-*Ptdss2*, whereas these populations were dramatically reduced when the tumor cells simultaneously harbored both sg*ATP11b* and sg*Ptdss2* mutants (Figure 7, C and D), suggesting that increased PS induced by sg*ATP11b* can cause the accumulation of MDSCs in mammary tissues and inhibit the actions of effector cells in the adaptive immune system.

**Figure 8:**
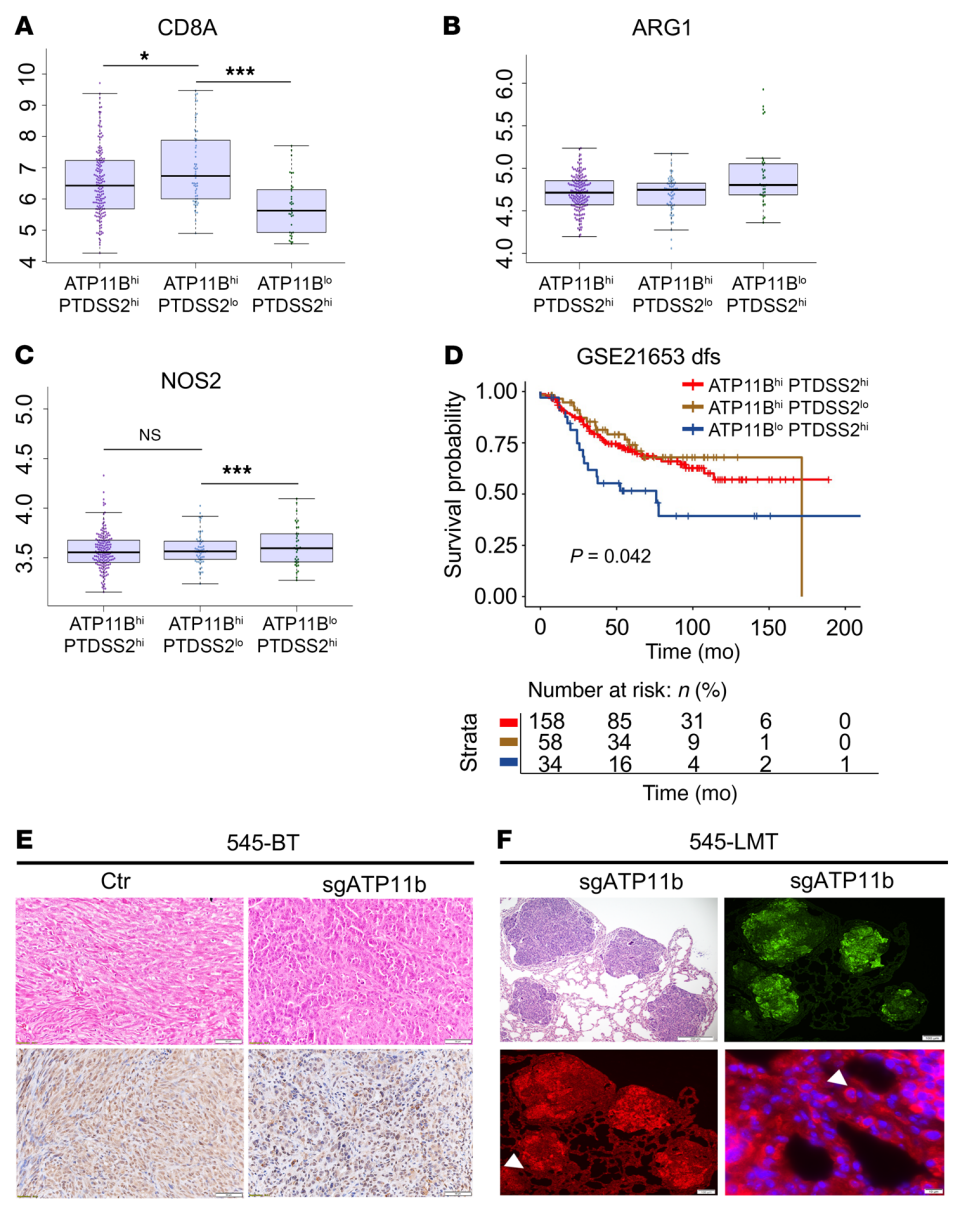
Nonapoptotic PS signal enhances immune suppression signal in Brca1/ATP11b double-mutant primary tumors. (**A**–**D**) Correlation of expression of CD8 (**A**), ARG1 (**B**), and NOS2 (**C**) in human breast cancer patients with different expression levels of *ATP11B* and *PTDSS2*, as indicated in the NCBI-GEO database (170 patients with *ATP11B*^hi^*PTDSS2*^hi^ expression, 59 patients with *ATP11B*^hi^*PTDSS2*^lo^ expression, and 35 patients with *ATP11B*^lo^*PTDSS2*^hi^ expression), and their survival outcomes (**D**) (GSE21653 data set). dfs, disease-free survival. (**E**) Images of sections of primary tumors obtained from 545-Ctr mice and sg*ATP11b*-545 mice stained by H&E and anti–TGF-β antibody (*n* = 5 mice per group). Scale bars: 50 μm. (**F**) Representative images of metastatic lungs by H&E, GFP, and TGF-β antibody (bottom panels) from sg*ATP11b*-545 mice (*n* = 3 mice per group). Scale bars: 200 μm (upper left), 100 μm (upper right and lower left), 10 μm (lower right). Statistical data in **A**–**C** were analyzed by 1-way ANOVA with Bonferroni’s multiple-comparison test; statistical data in **D** were analyzed by log rank test; data are presented as mean ± SEM. *P < 0.05, ***P < 0.001.

To test whether the accumulation of MDSCs caused by constitutive exposure to PS on the outer leaflet of the cell membrane of sg*ATP11b*-545 cells in primary tumors creates a stronger immunosuppressive signal for cancer metastasis, we studied the proliferation of T cells isolated from the spleen of WT mice, stimulated with CD3/CD28, and cocultured with MDSCs. While CD3/CD28 enhanced T cell proliferation (Figure 7E and Figure 8A), MDSCs isolated from the spleen of tumor-bearing mice implanted with parental 545 or sg*ATP11b*-545 cells partially or completely inhibited T cell proliferation (Figure 7, E and F). A similar inhibition was also observed with MDSCs isolated from the bone marrow of nude mice with the same genotype as those in the spleen cell experiment (Supplemental Figure 6, A and B). These data demonstrated that MDSC accumulation caused by an increased PS population on the cell membrane inhibits T cell proliferation and establishes a tumor-permissive microenvironment. Consistent with studies with our mouse model, analysis of the NCBI-GEO data set with information on breast cancer patients with *ATP11B*^lo^*PTDSS2*^hi^ expression revealed a strong correlation between the low expression of *CD8* and the high expression of *ARG1* and *NOS2*, which are highly expressed in MDSC populations (Figure 8, A–C). Breast cancer patients with this combination of gene expression had poor survival outcomes (Figure 8D).

To demonstrate that tumor cells with increased nonapoptotic PS had a stronger effect on the accumulation of MDSCs, we first examined the primary tumor and lung of recipient mice at days 3 and 21 with the implantation of 628 cells and sg*ATP11b*/OE-*Ptdss2*-628 cells. Increased MDSCs were observed by S100A9 staining in the primary tumor (Supplemental Figure 6, C and D) and the lung (Supplemental Figure 6, E and F) of 628 mice and sg*ATP11b*/OE-*Ptdss2*-628 mice compared with control mice (WT-Ctr). Very few MDSCs were observed in the WT-Cre mice at both time points, whereas the number of MDSCs was gradually increased in 628 and sg*ATP11b*/OE-*Ptdss2*-628 mice with a higher percentage in the lung than in the mammary gland. We also designed a time course experiment, harvesting 0.5 mL blood from the hearts of mice with implantation of WT-Ctr, 628, and sg*ATP11b*/OE-*Ptdss2*-628 cells at days 3, 7, 10, 14, 17, and 21 and comparing the dynamic changes of their MDSCs. Our data indicated that the percentage of MDSCs in both mice with 628 cells and mice with sg*ATP11b*/OE-*Ptdss2*-628 cells was higher than that in WT-Ctr mice at all the time points, suggesting that the MDSCs were already increased at early stages after tumor implantation (Supplemental Figure 6G).

Next, we examined circulating tumor cells (CTCs) in mice with implantation of 628 and sg*ATP11b*/OE-*Ptdss2*-628 cells. Because CTCs are quite rare and difficult to quantify, we used specific PCR primers for detecting cancer cells in the blood. We identified CTCs from 2 of 5 mice in the sg*ATP11b*/OE-*Ptdss2*-628 group at day 4 after the fat pad implantation but not in WT-Ctr– and 628-implanted mice. The number of mice with CTCs continued to increase at the following time points as more tumor cells entered the circulation, and CTCs could be detected in all 5 mice at day 11 (Supplemental Figure 6H). Altogether, these data indicate that tumor cells with high levels of PS could trigger the systemic response of the host immune system at early stages of tumor development that facilitates their metastasis.

To demonstrate that accumulation of MDSCs caused by nonapoptotic PS produces inhibitory signals that contribute to tumor metastasis, we next examined markers for M2-like macrophages and TGF-β signaling in tumor tissues of Ctr-545 and sg*ATP11b*-545 mice by immunohistochemistry, immunofluorescence, and Western blotting. The data revealed that the TGF-β signal was much stronger in the primary breast tumor tissues of the sg*ATP11b*-545 mice than in the control-545 mice (Figure 8E) and lung metastatic tissues (Figure 8F). The enhancement of the TME in the mice bearing tumors comprising sg*ATP11b*-545-GFP cells was further supported by the colocalization of TGF-β with CD206 or CD168 (Supplemental Figure 6I) by immunofluorescence and increased TGF-β signaling by Western blot analysis (Supplemental Figure 6J). These data demonstrate that constitutive exposure to PS in tumor cells creates a permissive TME that benefits cancer metastasis.

### Reversal of ATP11B^lo^PTDSS2^hi^ status by combinatory treatment with anti-PS/paclitaxel/docetaxel blocks breast cancer metastasis.

**Figure 9:**
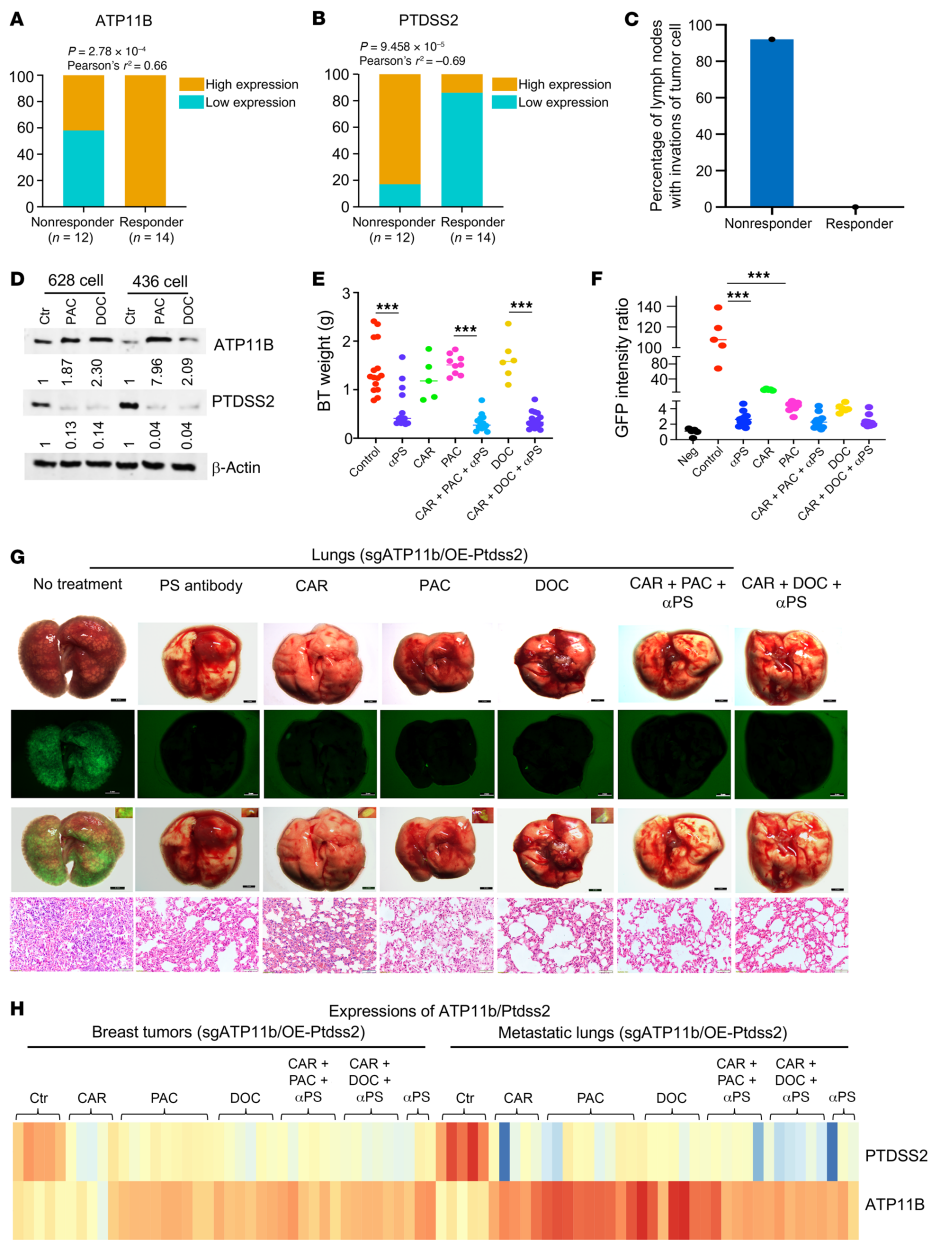
Inhibition of breast cancer metastasis with combination drug treatment. (**A** and **B**) Percentage of breast cancer patients with high or low *ATP11B* (**A**) and *PTDSS2* (**B**) expression in the nonresponder (*n* = 12) and responder (*n* = 14) groups. (**C**) Percentage of patients with infiltration of tumor cells into lymph nodes from the same cohort of patients in **A**. (**D**) Protein levels of *ATP11B* and *PTDSS2* in 628 and MDA-MB-436 cells after treatment with paclitaxel (PAC) or docetaxel (DOC) at a concentration of 2.4 nM for 5 days (*n* = 3). (**E**) Weights of tumors caused in sg*ATP11b*/OE-*Ptdss2*-628 cells under various conditions including control (CON), anti-PS antibody (αPS), carboplatin (CAR), PAC, CAR+PAC+αPS, DOC, and CAR+DOC+αPS at day 21. The concentration for each drug was 5 mg/kg (body weight, 20–22 g). For single treatment, the drugs were administrated every other day. For combination treatment, the drugs were administrated twice a week, and PS antibody was given at day 7 and day 14 (200 μg per mouse) (*n* = 6–15 mice per group). (**F** and **G**) Quantification of GFP intensities (**F**) and representative images (**G**) with BF, GFP intensity, overlap of BF and GFP, and H&E-stained sections of lungs for mice in **E** (*n* = 6–15 mice per group). Scale bars: 2 mm; (3 mm, No treatment). (**H**) Heatmap of gene expression of *ATP11b* and *Ptdss2* in breast tumor and lung tissues from the same cohort of mice in **E**–**G **(*n* = 6–15 mice per group). Statistical data in **E **and** F** were analyzed by 1-way ANOVA with Bonferroni’s multiple-comparison test; data are presented as mean ± SEM. ***P < 0.001.

Our data showed that the *ATP11b*^lo^*Ptdss2*^hi^ genotype was correlated with enhanced breast cancer metastasis. We hypothesized that metastasis might be minimized by drugs that can change gene expression by decreasing *ATP11B*^lo^*PTDSS2*^hi^ expression and increasing *ATP11B*^hi^*PTDSS2*^lo^ expression. Therefore, we searched various databases to identify whether any drugs can have effects on the expression of *ATP11B* and *PTDSS2* during the treatment of patients with breast cancer. After searching the TCGA and GEO data sets, we found that most drug-responsive patients (responders) exhibited an expression pattern of *ATP11B*^hi^*PTDSS2*^lo^, whereas most nonresponders showed the opposite expression pattern upon treatment with either paclitaxel (PAC) or docetaxel (DOC) (Supplemental Figure 7, A–D). To further explore the link of these expression patterns with patient metastatic status, we investigated a cohort of patients taking docetaxel treatment, including 12 nonresponders and 14 responders, by examining their protein levels of ATP11B, PTDSS2, and N-cadherin. We found that all 14 responders exhibited the ATP11B^hi^PTDSS2^lo^ expression pattern, the majority (12/14) showed low expression of N-cadherin, and none showed tumor cell infiltration into their lymph nodes (Figure 9, A and B). In contrast, mixed expression patterns of these genes were observed in the nonresponders, and most nonresponders (11/12) had tumor cell infiltration into their lymph nodes (Figure 9C) and expressed either high ATP11B or high N-cadherin protein levels (Supplemental Figure 7E and Supplemental Table 7), showing that they were not sensitive to drug treatment. These observations suggest either that, in the responder group, treatment with these drugs may reverse the expression of these 2 genes, or that patients with an ATP11B^hi^PTDSS2^lo^ expression pattern are sensitive to drug treatment. To confirm these possibilities, we examined whether treatment with PAC or DOC could induce changes in the levels of these 2 proteins in 628 and MDA-MB-436 cells. We found that protein levels of these 2 genes could be reversed after treatment with either PAC or DOC in both of these cell lines (Figure 9D).

To explore the possibility that the reversal of gene expression could block breast tumor metastasis, we first tested monotreatment of PAC, DOC, or anti-PS antibody (αPS) and then combined these treatments to study the effects on both tumor growth and tumor metastasis in the lung. Since carboplatin (CAR) is a common drug that can reduce primary tumor growth, we also included CAR in the drug treatment to our *ATP11b*^lo^*Ptdss2*^hi^ model mice. The data revealed that monotreatment with these 4 compounds separately did not reduce the weight of primary tumors, except for the PS antibody treatment (Figure 9E and Supplemental Figure 7, F–H), and the combination of CAR, PAC, and αPS or CAR, DOC, and αPS reduced the size of the primary tumors (Figure 9E and Supplemental Figure 7, F–H), suggesting that the combination treatment might contribute to reducing and delaying the progression of metastasis. Notably, the GFP metastatic signals in the lung were greatly reduced in all the monotreatment groups, and the GFP signals were reduced much more by the combined treatment administered to mice with *ATP11b*^lo^*Ptdss2*^hi^ tumors compared with the nontreatment group, which had a widespread strong GFP signal (Figure 9, F and G). In all cases, we observed improved lung structures (Figure 9F and Supplemental Figure 7I) but no apparent effect on body weight or spleen or liver volume (Supplemental Figure 7, J–L).

**Figure 10:**
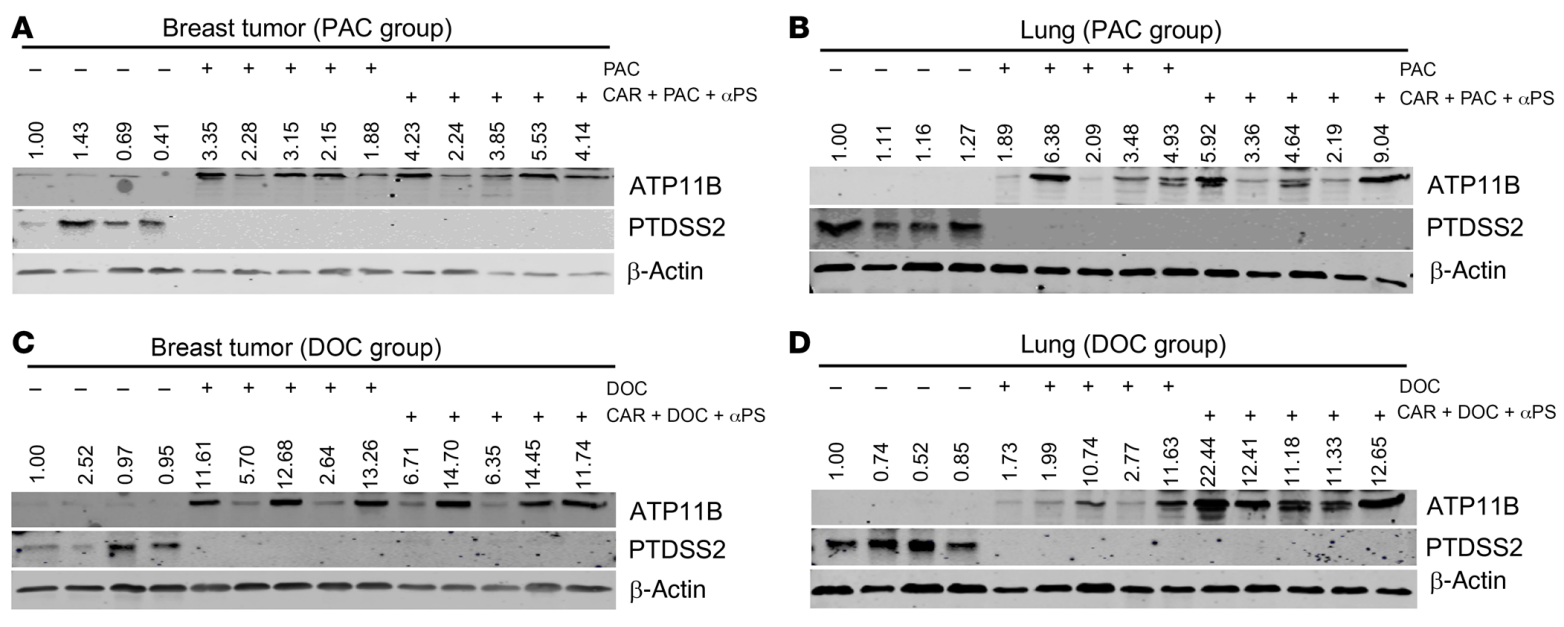
Reversal of ATP11B and PTDSS2 by combinatory treatment at protein level. (**A** and **B**) Protein levels of ATP11B and PTDSS2 in breast tumors (**A**) and lungs (**B**) treated with PAC and CAR + PAC + αPS antibody combination (*n* = 4–5 mice per group). (**C** and **D**) Protein levels of ATP11B and PTDSS2 in breast tumors (**C**) and lungs (**D**) treated with DOC and CAR + DOC + αPS antibody combination (*n* = 4–5 mice per group).

To determine whether the expression pattern of *ATP11b*^lo^*Ptdss2*^hi^ was changed to *ATP11b*^hi^*Ptdss2*^lo^ as determined at the gene expression and protein levels after drug treatment, we first analyzed mRNA levels of *ATP11b* and *Ptdss2* by qPCR. We found that both the single treatment and the combination treatment reversed *ATP11b*^lo^*Ptdss2*^hi^ expression to *ATP11b*^hi^*Ptdss2*^lo^ in breast and lung tissues in sg*ATP11b*/OE-*Ptdss2*-628 mice (Figure 9H). This observation was confirmed at the protein level in both the primary tumors and lungs in the same cohort of mice (Figure 10, A–D). Thus, our data demonstrated that PAC or DOC treatment reduced the gene expression pattern of *ATP11b*^lo^*Ptdss2*^hi^ and increased that of *ATP11b*^hi^*Ptdss2*^lo^ in vivo at both the mRNA and protein levels. The triple-combination treatment not only reduced the primary tumors but also greatly inhibited their metastasis.

## Discussion

In the present study, by CRISPR/Cas9–mediated genome-wide screening, we identified *ATP11b* as a potent suppressor of tumor metastasis. Our molecular analysis demonstrates that the expression signature of *ATP11B*^lo^*PTDSS2*^hi^ exposes PS on the outer leaflet of the cell membrane, which serves as a global immunosuppressive signal and synergizes with *BRCA1* deficiency in breast cancer metastasis. At the same time, our data also indicate that *ATP11B*^lo^*PTDSS2*^hi^ expression promotes tumor metastasis in *BRCA1*-WT tumors. These data illustrate the underlying mechanism involving the *ATP11B*/*PTDSS2* axis in tumor metastasis and provide a new selective therapeutic strategy for breast cancer patients with metastasis.

*ATP11b* acts as a potent metastatic repressor in multiple organs in *Brca1*-MT mice. A comparative study indicated that *BRCA1*-deficient breast cancer (*n* = 15) exhibited a high frequency of metastasis to the lung (60%) and brain (67%) compared with *BRCA1*-proficient breast cancers (*n* = 58) (38% and 10% in the lung and brain, respectively; ref. 15). However, a recent study of a larger cohort containing 46 *BRCA1* mutation carriers and 71 noncarriers indicated that *BRCA1* mutation status was not an independent predictor of distant metastasis, after adjusting for age and stage, although a higher rate of brain relapse in *BRCA1* mutation carriers than in noncarriers (58% vs. 24%) was observed (33). A more recent study, also with a larger patient cohort (30 *BRCA1* mutation carriers and 270 noncarriers) than the first study, confirmed that *BRCA1* mutation carriers frequently experienced a significantly higher metastatic incidence in the lung (15/30; 50%) than noncarriers (lung: 94/270, 35%), whereas brain metastasis might be attributed to the higher incidence of TNBC in *BRCA1* mutation carriers than noncarriers (14). These studies indicate that although *BRCA1* mutation seems to be associated with higher metastasis in some organs, the causal relationship between the mutation and cancer metastasis is complex and may be influenced by many factors. In this regard, although our function-based CRISPR/Cas9 screening identified many factors that might act as potentially metastatic suppressors, we first focused on *ATP11B*, which is the only factor whose disruption was found in all 5 metastatic organs, i.e., the lung, liver, spleen, brain, and kidney.

*ATP11B* and its closely related family members, *ATP11A* and *ATP11C*, belong to a subclass (class 6) of P4-ATPases that serve as lipid flippases critical for the asymmetrical distribution of PS on the inner leaflet of the cell membrane (25). This asymmetrical PS accumulation in the inner membrane plays some very important roles, such as the maintenance of membrane curvature and fluidity, membrane-cytoskeletal anchoring and membrane trafficking, and proper localization and/or activation of some proteins, including the E3 ubiquitin ligase NEDD4, protein kinase C isoforms, and phosphatase and tensin homolog–deleted on chromosome 10 (PTEN) (34–36). In general, the absence of PS on the outer leaflet of the plasma membrane is critical for cell viability, since exposure of PS on the cell surfaces is an “eat me” signal that induces phagocytosis during apoptosis (37, 38). It was shown that the activation of caspase leads to the activation of Xkr8, a scramblase that breaks membrane asymmetry by randomizing all phospholipid species between leaflets, which effectively increases the accumulation of PS on the external side of the membrane (37–39). PS externalization is also observed in the maturation and/or activation of some specific types of cells, such as platelets during coagulation and platelet aggregation, mature macrophages, and dendritic cells (23). Furthermore, in an experimental system, externalization of PS by TMEM16F, which is activated by Ca^2+^, did not trigger engulfment by macrophages (40). In our study, we found that the externalization of PS caused by ATP11B disruption also did not induce apoptosis, as revealed by TUNEL assay performed with an APO-BrdU Kit and Western blot analysis with the anti–caspase-3 antibody, even though these cells are positive for annexin V, which binds to PS on the outer membrane.

ATP11B^lo^ and PTDSS2^hi^ drive constitutive exposure of outer membrane PS to promote the metastasis of *Brca1*-MT tumors. PS is produced from phosphatidylcholine through *Ptdss1* and from phosphatidylethanolamine through *Ptdss2*; both *Ptdss1* and *Ptdss2* serve as catalytic enzymes for PS synthesis (29, 30). Because the PTDSS2 protein level was stably elevated in both *Brca1*-MT mammary gland and tumor tissues in *Brca1*-MT mice, we conducted a promoter luciferase reporter assay, and the data revealed that the *Ptdss2* promoter is negatively regulated by *Brca1*. This finding indicated that *Brca1*-deficient cells can generate more PS than *Brca1*-WT cells. Moreover, we also found reduced expression of *ATP11b* in *Brca1*-MT mammary glands compared with WT glands, and it was even lower in tumor tissues, suggesting that *Brca1* might play a role in maintaining *ATP11b* expression. Thus, *Brca1* deficiency may result in the *ATP11b*^lo^ and *Ptdss2*^hi^ phenotype that facilitates externalization of PS, which may account for the higher rate of metastasis of *Brca1*-MT mammary tumors. In our CRISPR/Cas9–mediated genome-wide screening, disruption of *ATP11b* further enhanced exposure of PS to the outer leaflet of the cell membrane.

Our data demonstrated that the constitutive exposure of PS on the cell membrane caused by *ATP11B*^lo^ and *PTDSS2*^hi^ in *Brca1*-MT cancer is critical for triggering cancer metastasis. The promotion of cancer metastasis by high levels of PS has been studied extensively (24). It has been shown that the accumulation of PS on the extracellular leaflet of the plasma membrane may affect asymmetric membrane bilayers and trigger global immunosuppressive signaling, including increased production of TGF-β, which favors the formation of a tumor microenvironment (23, 41).

Combination therapy consisting of the anti-PS antibody with docetaxel or paclitaxel represses breast cancer metastasis. Most *BRCA1* mutation–related breast cancers are in the TNBC category, which is difficult to treat (6, 7). The use of the PARP inhibitor olaparib achieved a response rate of 59.9% in the treatment group compared with 28% in the standard therapy group (42). However, even in the responsive patients, olaparib only moderately extended life, from 4.2 months to 7 months on average, in comparison with the standard therapy group (42), which highlights the need for effective therapeutic treatment strategies for *BRCA1* mutation–associated breast cancer (43). In our efforts to identify drugs that might inhibit *BRCA1*-deficient tumor growth and metastasis, we found that paclitaxel or docetaxel treatment can reverse the expression pattern of *ATP11B* and *PTDSS2* in breast cancers and significantly inhibit cancer metastasis. Notably, monotreatment or dual treatment with these drugs does not have a strong impact on primary tumor formation unless an anti-PS antibody is also used. In this regard, it was recently shown that a chemical activator of *p53* in combination with an antibody that targets PS on tumor blood vessels and disrupts tumor vasculature effectively inhibits metastasis of *p53*-expressing TNBC cells to the lungs in a mouse model (44). Our study further illustrated that PS should serve as a target for cancer therapy, as it is an essential component of the cancer cell membrane and plays an important role in maintaining cancer cell viability and metastasis (45–47).

In summary, we performed a genome-wide screening of metastatic repressors and identified *ATP11b* as a potent metastasis suppressor for *Brca1*-deficient cancers, as its knockout enhances tumor metastasis in vivo. Mechanistically, its low expression or deficiency together with high expression of *Ptdss2* increased the PS population on the outer leaflet of the cell membrane and generated a global immunosuppressive signal, leading to cancer metastasis. Our data also highlight a critical role of PS in maintaining both growth and metastasis of cancer cells, as its blocking by anti-PS antibody affects both processes. Our further analysis revealed that in *Brca1*-WT cancer cells, *ATP11b* and *Ptdss2* signaling also plays similar roles in cancer metastasis. We demonstrated that *ATP11b*^lo^*Ptdss2*^hi^ expression can be reversed by the anticancer drugs docetaxel or paclitaxel and by combination therapy with an anti-PS antibody. The reversal of the *ATP11B*^lo^*PTDSS2*^hi^ pattern represses breast cancer growth and metastasis. This finding may facilitate the development of a selective treatment strategy for breast cancer patients to prevent breast cancer metastasis.

## Methods

### Cell lines.

The 545-GFP cell line was derived from *Brca1^Co/Co^ MMTV-Cre* (*Brca1*-MSK) mice, which develop only primary mammary tumors and have minimal metastatic ability, as determined by labeling with GFP. The 628-GFP cell line was also derived from *Brca1*-MSK mice, which not only develop primary mammary tumors but also develop multiple metastatic tumors in other organs, which were identified by the GFP label. B477 is a *Brca1*-WT mammary gland epithelial cell line, and G600 was derived from *Brca1*-MT mammary gland epithelial cells. T47D (catalog HTB-133), MDA-MB-231 (catalog CRM-HTB-26), MDA-MB-436 (catalog HTB-130), and HEK293T (catalog ACS-4500) cells were purchased from American Type Culture Collection (ATCC). All the cells above were cultured with high-glucose DMEM containing 10% FBS and antibiotics (100 μg/mL streptomycin and 100 U/mL penicillin; Gibco). All cell lines were cultured at 37°C in an atmosphere with 5% CO_2_. All other human breast cancer cell lines, including MDA-MB-436, MDA-MB-468, T47D, and HEK293T, were purchased from ATCC.

### sgRNA lentiviral library preparation, viral infection, and labeling of GFP cell lines.

Lentiviruses were generated according to the second-generation lentiviral packaging system using a standard procedure. The titer of the virus was confirmed by the infection of 293T cells. GeCKOv2 sgRNA from Addgene was packaged into lentiviral particles. 545 cells (minimally invasive metastatic breast tumor cells) and 628 cells (highly metastatic breast tumor cells) were infected with lenti-GFP viruses at an MOI of 10 for 48 hours, and GFP-positive cells were isolated by FACS and continued to grow. 545-GFP cells were infected with the lentiviral GeCKOv2 sgRNA library at an MOI of 0.1 for 48 hours, and the transfected cells were grown in normal medium (DMEM plus 10% FBS) for 48 hours after the infected cells were selected with puromycin for 7 days. The transfected cells were then implanted into the mammary fat pads of nude mice with 1 × 10^–6^ cells per fat pad, and 5 million cells were saved as a control. Primary breast tumors and metastatic tumors in the lung, liver, kidney, spleen, and brain were collected 8 weeks after injection of the cells transfected with the GeCKOv2 sgRNA library.

### Validation of metastatic tumor suppressors.

Oligonucleotide sequences of candidate genes identified in the next-generation sequencing analysis were cloned into the lenti-v2 (vector 2), and the individual oligonucleotide sequence for sgRNA was packaged. 545-GFP cells infected with lentiviral particles containing the candidate genes were injected into the mammary fat pads of nude mice after puromycin selection for 7 days. Primary tumors and metastatic tumors from the lung, liver, brain, kidney, and spleen were harvested 8 weeks after the injection. DNA from the tumor tissues was extracted, and Sanger sequencing was performed to confirm the indels produced by the specific sgRNAs. Total RNA was extracted using TRIzol solution in a Precellys Evolution tissue homogenizer, and the purity and concentration of the RNA were measured with a NanoDrop 8000 spectrophotometer (Thermo Fisher Scientific). cDNA was reverse-transcribed from qualified total RNA extracted from tumor tissues using a QuantiTect Reverse Transcription kit. Gene expression was determined using a QuantStudio 7 Flex Real-Time PCR system with a SYBR-Green kit (catalog RR820A, TaKaRa company), and the relative expression of mRNA was calculated by the 2^–ΔΔCt^ method.

### Detailed in vivo and in vitro studies and in silico analysis.

See Supplemental Methods for detailed virus preparation, in vivo and in vitro studies, and analysis of next-generation sequencing data.

### Data availability.

The RNA-Seq data generated in this study were deposited in the NCBI’s Sequence Read Archive under accession number PRJNA753220.

### Statistics.

All the statistical analysis in this study was carried out with R 3.4.3 software. For the comparison of multiple groups, 1-way ANOVA with Bonferroni’s multiple-comparison test was used. Comparison of 2 groups composed of continuous data (except next-generation sequencing data) was analyzed by 2-tailed Student’s *t* test. Survival of patients in 2 groups was analyzed by log rank test. *P < 0.05; **P < 0.01; ***P < 0.001. *P* values lower than 0.05 were considered significant.

### Study approval.

All mouse strains were maintained in the animal facility of the Faculty of Health Science, University of Macau, according to institutional guidelines. All experiments were approved by the Animal Ethics Committees of the Faculty of Health Science, University of Macau (protocol UMARE-AMEND-100).

## Author contributions

JX, CXD, and XX conceived and designed the study. JX, SMS, XS, J Li, J Liu, LM, YW, TA, and JHL developed methodology. JX, XZ, SMS, and UIC acquired data (provided animals, acquired and managed patients, provided facilities, etc.). JX, RA, KM, CXD, and XX analyzed and interpreted data (e.g., statistical analysis, biostatistics, computational analysis). JX, XX, and CXD wrote, reviewed, and/or revised the manuscript.

## Supplementary Material

Supplemental data

Supplemental table 1

Supplemental table 2

Supplemental table 3

Supplemental table 4

Supplemental table 5

Supplemental table 6

Supplemental table 7

## Version 1 01/13/2022

In-Press Preview

## Version 2 03/01/2022

Electronic publication

## References

[1] Torre LA (2015). Global cancer statistics, 2012. CA Cancer J Clin.

[2] Siegel RL (2021). Cancer statistics, 2021. CA Cancer J Clin.

[3] Apostolou P, Fostira F (2013). Hereditary breast cancer: the era of new susceptibility genes. Biomed Res Int.

[4] Hall MJ (2009). BRCA1 and BRCA2 mutations in women of different ethnicities undergoing testing for hereditary breast-ovarian cancer. Cancer.

[5] Sharma B (2018). BRCA1 mutation spectrum, functions, and therapeutic strategies: the story so far. Curr Probl Cancer.

[6] Spurdle AB (2014). Refined histopathological predictors of BRCA1 and BRCA2 mutation status: a large-scale analysis of breast cancer characteristics from the BCAC, CIMBA, and ENIGMA consortia. Breast Cancer Res.

[7] Bauer KR (2007). Descriptive analysis of estrogen receptor (ER)-negative, progesterone receptor (PR)-negative, and HER2-negative invasive breast cancer, the so-called triple-negative phenotype: a population-based study from the California cancer Registry. Cancer.

[8] Xu X (1999). Conditional mutation of Brca1 in mammary epithelial cells results in blunted ductal morphogenesis and tumour formation. Nat Genet.

[9] Xu X (2001). Genetic interactions between tumor suppressors Brca1 and p53 in apoptosis, cell cycle and tumorigenesis. Nat Genet.

[10] Xu X (1999). Centrosome amplification and a defective G2-M cell cycle checkpoint induce genetic instability in BRCA1 exon 11 isoform-deficient cells. Mol Cell.

[11] Chen Q (2020). BRCA1 deficiency impairs mitophagy and promotes inflammasome activation and mammary tumor metastasis. Adv Sci (Weinh).

[12] Miao K (2020). NOTCH1 activation compensates BRCA1 deficiency and promotes triple-negative breast cancer formation. Nat Commun.

[13] Liu J (2020). Characterization of BRCA1-deficient premalignant tissues and cancers identifies Plekha5 as a tumor metastasis suppressor. Nat Commun.

[14] Song Y (2020). Patterns of recurrence and metastasis in BRCA1/BRCA2-associated breast cancers. Cancer.

[15] Albiges L (2005). Spectrum of breast cancer metastasis in BRCA1 mutation carriers: highly increased incidence of brain metastases. Ann Oncol.

[16] Lambert AW (2017). Emerging biological principles of metastasis. Cell.

[17] Birkbak NJ, McGranahan N (2020). Cancer genome evolutionary trajectories in metastasis. Cancer Cell.

[18] Schrijver W (2018). Mutation profiling of key cancer genes in primary breast cancers and their distant metastases. Cancer Res.

[19] Guo S, Deng CX (2018). Effect of stromal cells in tumor microenvironment on metastasis initiation. Int J Biol Sci.

[20] Dysthe M, Parihar R (2020). Myeloid-derived suppressor cells in the tumor microenvironment. Adv Exp Med Biol.

[21] Pashayan N (2020). Personalized early detection and prevention of breast cancer: ENVISION consensus statement. Nat Rev Clin Oncol.

[22] Karthikeyan S (2021). Hierarchical tumor heterogeneity mediated by cell contact between distinct genetic subclones. J Clin Invest.

[23] Birge RB (2016). Phosphatidylserine is a global immunosuppressive signal in efferocytosis, infectious disease, and cancer. Cell Death Differ.

[24] Skotland T (2020). The role of lipid species in membranes and cancer-related changes. Cancer Metastasis Rev.

[25] Andersen JP (2016). P4-ATPases as phospholipid flippases—structure, function, and enigmas. Front Physiol.

[26] Pomorski T, Menon AK (2006). Lipid flippases and their biological functions. Cell Mol Life Sci.

[27] Suzuki J (2013). Xk-related protein 8 and CED-8 promote phosphatidylserine exposure in apoptotic cells. Science.

[28] Calianese DC, Birge RB (2020). Biology of phosphatidylserine (PS): basic physiology and implications in immunology, infectious disease, and cancer. Cell Commun Signal.

[29] Vance JE, Tasseva G (2013). Formation and function of phosphatidylserine and phosphatidylethanolamine in mammalian cells. Biochim Biophys Acta.

[30] Farine L (2017). Phosphatidylserine synthase 2 and phosphatidylserine decarboxylase are essential for aminophospholipid synthesis in Trypanosoma brucei. Mol Microbiol.

[31] Meszaros B (2018). IUPred2A: context-dependent prediction of protein disorder as a function of redox state and protein binding. Nucleic Acids Res.

[32] Brodie SG (2001). Multiple genetic changes are associated with mammary tumorigenesis in Brca1 conditional knockout mice. Oncogene.

[33] Paluch-Shimon S (2016). Neo-adjuvant doxorubicin and cyclophosphamide followed by paclitaxel in triple-negative breast cancer among BRCA1 mutation carriers and non-carriers. Breast Cancer Res Treat.

[34] Lemmon MA (2008). Membrane recognition by phospholipid-binding domains. Nat Rev Mol Cell Biol.

[35] Eustace NJ (2019). Myristoylated alanine-rich C-kinase substrate effector domain phosphorylation regulates the growth and radiation sensitization of glioblastoma. Int J Oncol.

[36] Plant PJ (1997). The C2 domain of the ubiquitin protein ligase Nedd4 mediates Ca2+-dependent plasma membrane localization. J Biol Chem.

[37] Fadok VA (2001). Loss of phospholipid asymmetry and surface exposure of phosphatidylserine is required for phagocytosis of apoptotic cells by macrophages and fibroblasts. J Biol Chem.

[38] Segawa K, Nagata S (2015). An apoptotic ‘eat me’ signal: phosphatidylserine exposure. Trends Cell Biol.

[39] Kimani SG (2014). Contribution of defective PS recognition and efferocytosis to chronic inflammation and autoimmunity. Front Immunol.

[40] Suzuki J (2013). Calcium-dependent phospholipid scramblase activity of TMEM16 protein family members. J Biol Chem.

[41] Skotland T (2017). Molecular lipid species in urinary exosomes as potential prostate cancer biomarkers. Eur J Cancer.

[42] Robson M (2017). Olaparib for metastatic breast cancer in patients with a germline BRCA mutation. N Engl J Med.

[43] Franzoi MA (2021). Evidence-based approaches for the management of side-effects of adjuvant endocrine therapy in patients with breast cancer. Lancet Oncol.

[44] Liang Y (2019). APR-246 alone and in combination with a phosphatidylserine-targeting antibody inhibits lung metastasis of human triple-negative breast cancer cells in nude mice. Breast Cancer (Dove Med Press).

[45] Szlasa W (2020). Lipid composition of the cancer cell membrane. J Bioenerg Biomembr.

[46] Chang W (2020). Targeting phosphatidylserine for cancer therapy: prospects and challenges. Theranostics.

[47] Sharma B, Kanwar SS (2018). Phosphatidylserine: a cancer cell targeting biomarker. Semin Cancer Biol.

