# Supplementary material for: ATP11B inhibits breast cancer metastasis in a mouse model by suppressing externalization of nonapoptotic phosphatidylserine
Source: J Clin Invest. 2022 Mar 1;132(5):e149473. doi: 10.1172/JCI149473 (PMC8884903; doi:10.1172/JCI149473)
Supplement: Supplemental data [file jci-132-149473-s121.pdf]

ATP11B inhibits breast cancer metastasis in a mouse model by suppressing externalization of non-apoptotic phosphatidylserine

Jun Xu[1,2], Sek Man Su[1,2], Xin Zhang[1,2], Un In Chan[1,2], Ragini Adhav[1,2], Xiaodong Shu, [1,2], Jianlin Liu[1,2], Jianjie Li[1,2], Lihua Mo [1,2], Yuqing Wang[1,2], Tingting An[1,2], Josh haipeng lei[1,2], Kai Miao[1,2,3], Chu-Xia Deng[1,2,3] *, Xiaoling Xu[1,2,3] *

[1]Cancer Centre, Faculty of Health Sciences, University of Macau, Macau SAR, China.

[2]Centre for Precision Medicine Research and Training, Faculty of Health Sciences, University of Macau, Macau SAR, China

[3]MOE Frontier Science Centre for Precision Oncology, University of Macau, Macau SAR, China

One Sentence Summary: ATP11B^{low}/PTDSS2^{high} Axis promotes metastasis in breast cancer

Conflict of interests: The authors declare no conflict of interest

***Corresponding author:** Chu-Xia Deng & Xiaoling Xu

***Correspondence:** cxdeng@um.edu.mo ; xiaolingx@um.edu.mo

Cancer Center
Faculty of Health Sciences
University of Macau
Macau, Macau, SAR, China
Center for Precision Medicine Research and Training
University of Macau
Macau, Macau, SAR, China
E-mail: cxdeng@um.edu.mo ; xiaolingx@um.edu.mo

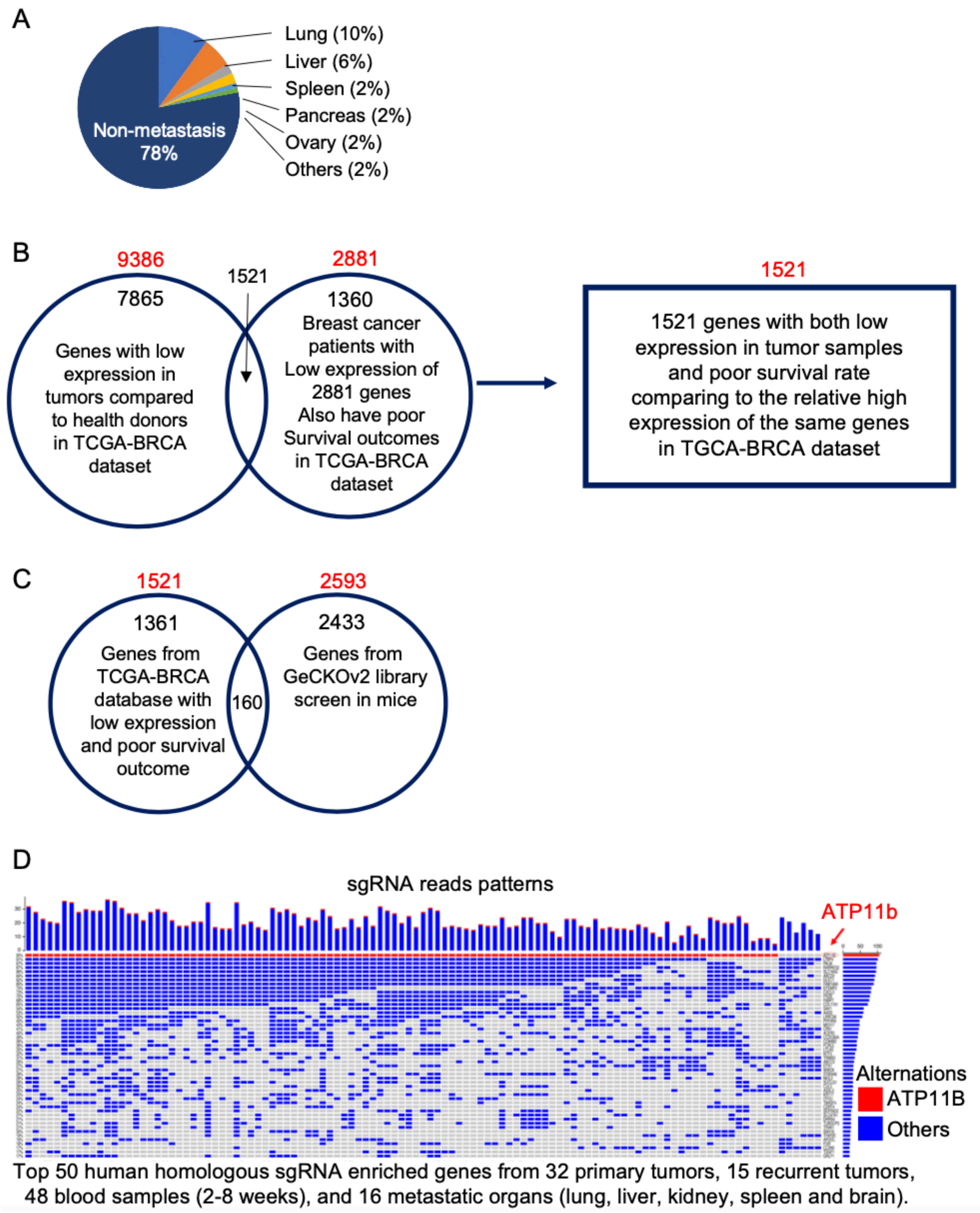

Figure S1. Potential tumor metastatic suppressor genes.

(A) Percentage of mice with metastasis from 207 *Brca1*-MSK (*Brca1*$^{Co/Co}$*;MMTV-Cre*) mice with breast tumors presented by pie chart . **(B)** 1521 potential tumor suppressors of 9385 genes that have lower expression levels, compared to those of healthy donors, in breast cancer patients, and 2881 genes that not only express

lower levels compared to healthy donors ($P < 0.05$) but are also associated with poorer survival outcomes ($P < 0.05$) compared to the patients expressing the same genes at higher levels (supplementary table 2). The gene expression profiles are from 1100 breast cancer patients and 112 healthy donors with data in the TCGA-BRCA dataset. **(C)** 160 common genes identified from a comparison of both human candidate suppressor genes (1521) and mouse suppressor candidate genes (2594) upon screening the CRISPR library in vivo. **(D)** The sgRNA reads patterns of the top 50 of 1145 genes whose sgRNA reads counts were 10 or more compared to those in the cells before injection into nude mice, as determined by OncoPrint. The sgRNA reads were obtained from 32 primary tumors, 48 blood samples, 15 recurrent and 16 metastatic tumors during 8 weeks of breast tumor growth.

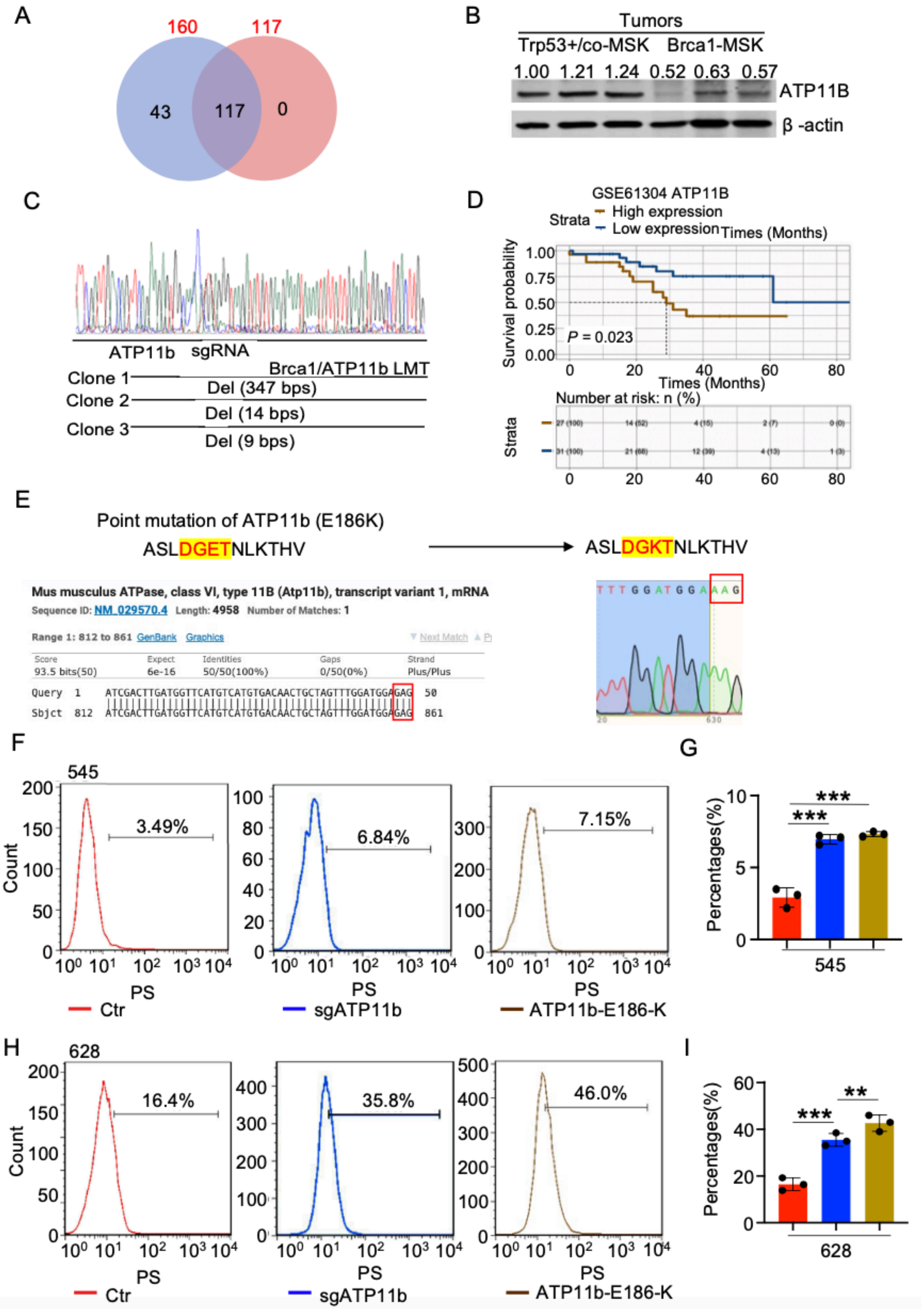

Figure S2. ***ATP11b*** **is associated with multiorgan metastasis.**

(A) Comparison of 160 human homologous tumor suppressor genes with 117 human homologous metastatic candidate genes. **(B)** The protein level of ATP11B

in mammary tumor tissues of *Trp53*-MSK and *Brca1*-MSK mice by Western blot analysis (n = 3 mice/group). **(C)** Representative images of Sanger sequencing of metastatic nodules in the lungs of three different individual mice. **(D)** The plot of survival outcome correlated with the expression of *ATP11B* in breast cancer patients (GSE61304 dataset, 58 patients were included). **(E)** DGET motif in ATP11B is mutated to DGKT (E186K) motif and viewed by NCBI blast and SnapGene Viewer. **(F-G)** PS displacement by FACS analysis with PS antibody on cell membrane of 545 parental cell (red), 545 cell with expression of sg*ATP11b* (blue), and *ATP11b*-E186K (brown). **(G)** Quantification PS displacement for panel **(F)** (n = 3 times). **(H)** PS displacement by FACS analysis with PS antibody on cell membrane of 628 parental cell (red), 628 cell with expression of sg*ATP11b* (blue), *ATP11b*-E186K (brown). **(I)** Quantification of PS displacement for panel **(H)** (n = 3 times). Statistical data in **D** were analyzed by Logrank test, **G** and **I** were analyzed by 1-way ANOVA with Bonferroni's multiple-comparison test, data were presented as mean ± SEM. **$P < 0.01$, ***$P < 0.001$.

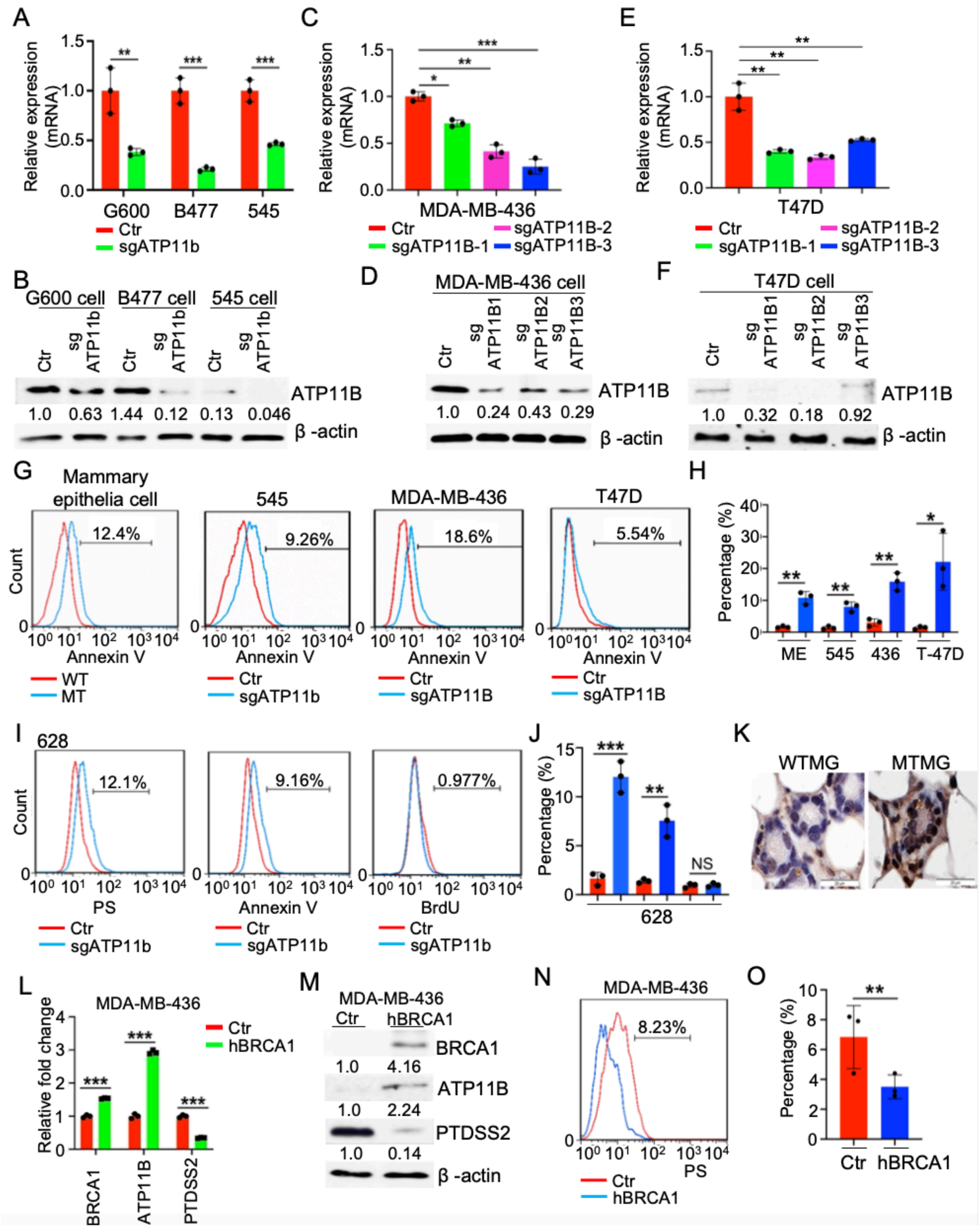

Figure S3. Increased nonapoptotic PS externalization in ATP11b^{low} cells.

(A-B) *ATP11b* mRNA expression (A) and protein levels (B) in *Brca1*-MT (G600) mammary epithelial cells, *Brca1*-WT (B477) mammary epithelial cells, and *Brca1*-MT low metastatic tumor cells (545) without or with expression of sg*ATP11b* in

three different mouse cell lines (*n* = 3). **(C-D)** *ATP11B* mRNA expression (C) and protein levels (D) in MDA-MB-436 cells expressing three different sgRNAs (*n* = 3). **(E-F)** *ATP11B* mRNA expression **(E)** and protein levels **(F)** in T47D cells expressing three different sgRNAs (*n* = 3). **(G-H)** PS displacement in both WT and *Brca1*-MT primary mammary epithelial cells, 545 cells, 628 cells, and MDA-MB-436 cells without or with the expression of sg*ATP11B* as detected with the anti-Annexin V antibody by FACS analysis **(G)** and quantification **(H)** for panel **(G)** (*n* = 3 times). **(I)** The PS displacement on the cell membrane of 628 cells without or with the expression of sg*ATP11B* as determined by FACS analysis with anti-PS antibody and anti-Annexin V antibody. The apoptotic cell population as determined with an APO-BrdU™ kit. **(J)** Quantification for panel **(I)** (*n* = 3 times). **(K)** The PS level in mammary epithelial cells of both WT and *Brca1*-MSK mice by IHC staining with an anti-PS antibody (*n* = 3 mice/group). **(L-M)** Expression of *BRCA1*, *ATP11B*, and *PTDSS2* at mRNA level **(L)** and protein levels (M) in MDA-MB-436 cells without (red) or with h*BRCA1* cDNA expression (*n* = 3). **(N-O)** PS displacement on the cell membrane in MDA-MB-436 cells without (red) or with (blue) the expression of h*BRCA1* cDNA by FACS analysis with PS antibody **(N)** and quantification **(O)** for panel **(N)** (*n* = 3 times). Error bars show mean ± SEM. Two-tailed student's t test was used to calculate significance. **P* < 0.05, ***P* < 0.01, ****P* < 0.001, NS no statistical significance.

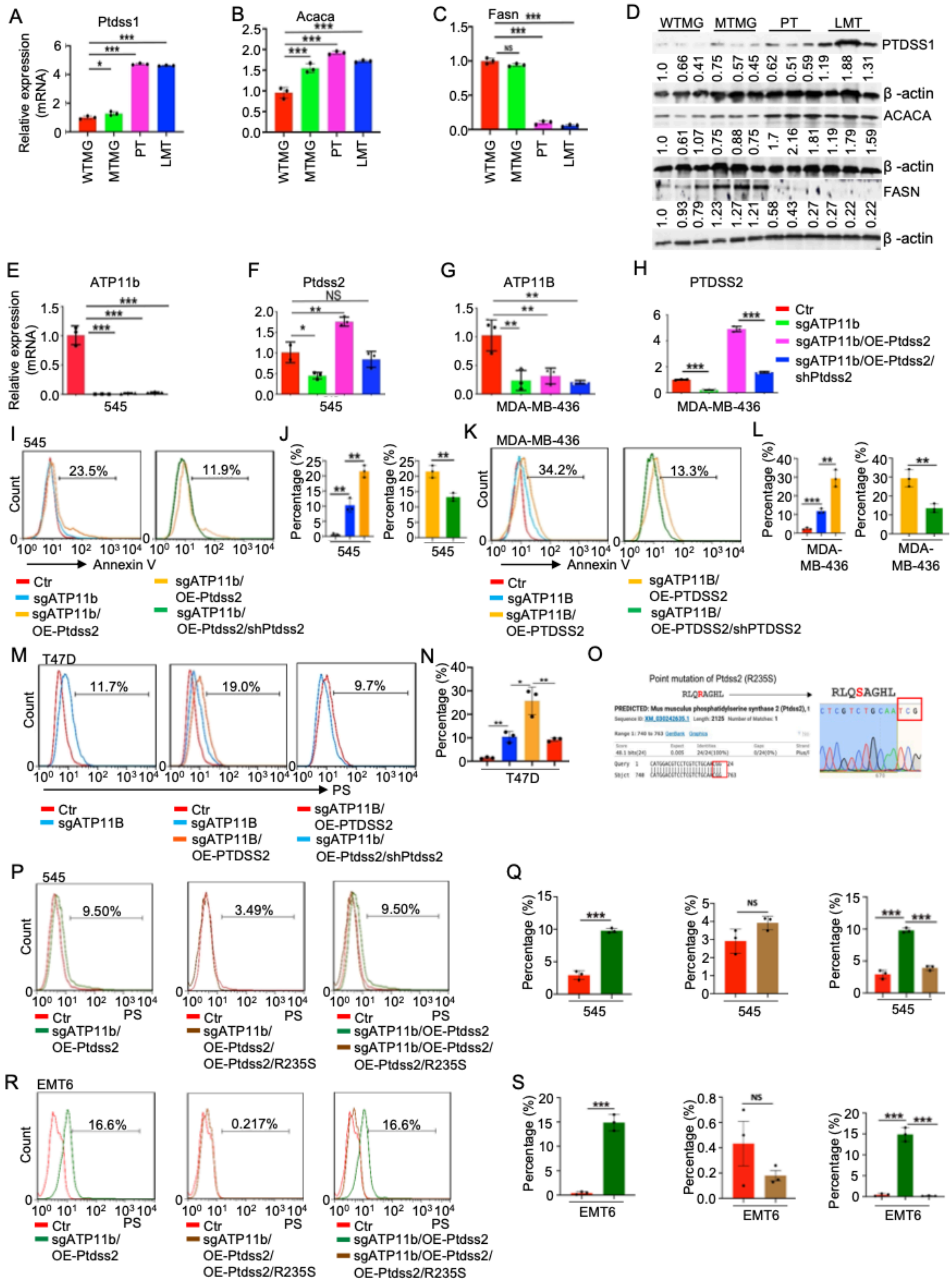

Figure S4. Elevated Ptdss2 increases PS on the outer leaflet of the cell membrane.

(A-D) Expressions at mRNA level of *Ptdss2* **(A)**, *Acaca* **(B)**, and *Fasn* **(C)** and protein levels of PTDSS2, ACACA, and FASN **(D)** in WTMG, MTMG, PT, and MT

from *Brca1*-MSK mice as determined by qPCR and Western blots (*n* = 3 or more mice/group). **(E-H)** Expression of *ATP11b* and *Ptdss2* in both 545 **(E-F)** and MDA-MB-436 cells **(G-H)** without or with the expression of sg*ATP11b*, sg*ATP11b*/OE-*Ptdss2*, or sg*ATP11b*/OE-*Ptdss2*/sh*Ptdss2*, as determined by qPCR (*n* = 3 times). **(I-L)** PS displacement on cell membrane of both 545 **(I)** and MDA-MB-436 **(K)** cells, respectively, without or with the expression of either sg*ATP11b*, or sg*ATP11b*/OE-*Ptdss2*, or sg*ATP11b*/OE-*Ptdss2*/sh*Ptdss2* by FACS analysis using and anti-Annexin V antibody and quantifications **(J)** for panel **(I)** and **(L)** for panel **(K)** (*n* = 3 times)**. (M-N)** PS displacement on the cell membranes of T47D cells without or with the expression of either sg*ATP11B*, overlap of sg*ATP11B* with sg*ATP11B*/OE-*PTDSS2*, and overlap of two plots as revealed by FACS analysis with PS antibody and quantification **(N)** for panel **(M)** (*n* = 3 times). **(O)** The R235S mutation of *PTDSS2* in mouse by NCBI blast and SnapGene Viewer**. (P-Q)** PS displacement on the cell membrane of 545 cells without, or with the expression of sg*ATP11b*/OE-*Ptdss2*, sg*ATP11b*/OE-*Ptdss2*/OE-*Ptdss2*-R235S mutation and overlap of two plots as determined by anti-PS antibody and quantification (**Q**) for panel **(P)** (*n* = 3 times). **(R-S)** PS displacement on the cell membrane of EMT6 cells without, or with the expression of sg*ATP11b*/OE-*Ptdss2*, sg*ATP11b*/OE-*Ptdss2*/OE-*Ptdss2*-R235S mutation and overlap of two plots by anti-PS antibody and quantification **(S)** for panel **(R)** (*n* = 3 times). Statistical data in **A-C**, **E**-**H, Q, S** were analyzed by 1-way ANOVA with Bonferroni's multiple-comparison test, rest of Statistical data were analyzed by two-tailed student's t test, data were presented as mean ± SEM. **P* < 0.05, ***P* < 0.01, ****P* < 0.001, NS no statistical significance.

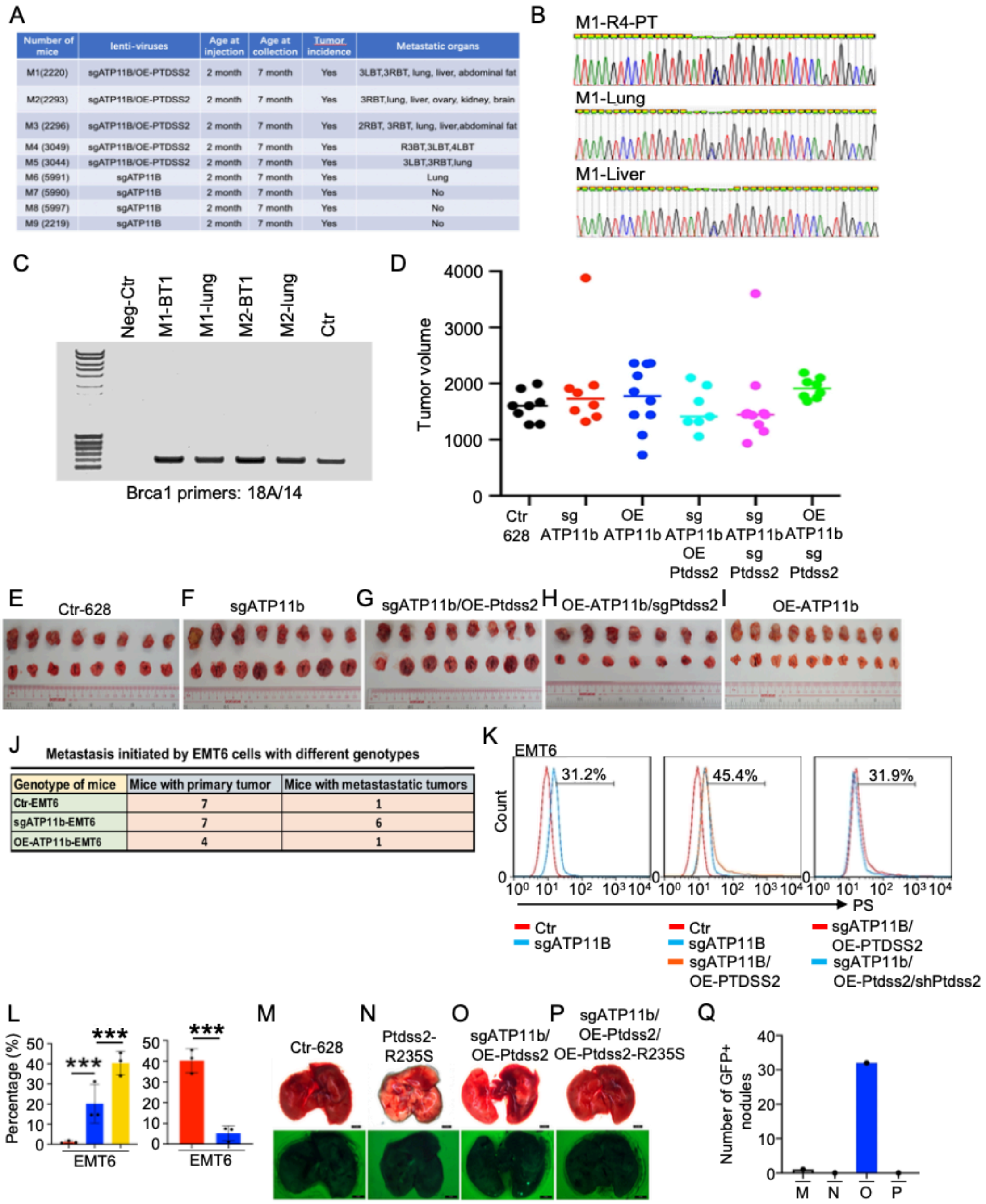

Figure S5. Cells expressing *ATP11b*^{Low}/*Ptdss2*^{high} enhance breast cancer metastasis

(A) The summary information of intraductal injections with lentivirus expressing sg*ATP11b* only (*n* = 4 mice), mixed sg*ATP11b* and OE-*Ptdss2*-GFP lentiviruses (*n* = 5 mice), and lentivirus vector only (*n* = 3 mice) to two-month-old (*Brca1*-*Trp53*-MSK) virgin mice. Primary and metastatic tumors were harvested after 5-month mammary ductal injection. **(B)** Representative indels from the primary tumors (R4-

PT), lungs, and livers of *Brca1-Trp53*-MSK mice with mammary intraductal injections revealed by Sanger sequences with *ATP11b* specific primers. **(C)** Identification of *Brca1* exon 11 deletions in both primary tumors and metastatic lungs by PCR with primers of *Brca1*-18A/14. **(D)** The plot of tumor volume four weeks after mammary fat pad implantation with 628 cells only (Ctr, black), cells with the expression of sg*ATP11B* (red), OE-*ATP11b* (dark blue), sg*ATP11b*/OE-*Ptdss2* (light blue), sg*ATP11b*/sg*Ptdss2* (pink), or OE-*ATP11b*/sg*Ptdss2* (green) (*n* = 7 or more mice/group). **(E-I)** Representative images of primary tumors and lungs from 628-Ctr mice **(E)**, sg*ATP11b*-628 mice **(F)**, sg*ATP11b*/OE-*ATP11b*-628 mice **(G)**, sg*ATP11b*/OE-*Ptdss2*-628 **(H)**, and OE-*ATP11b*-628 mice **(I)** (*n* = 7-10 mice/group). **(J)** Metastatic status of EMT6 cells expressing sg*ATP11b* in Balb/c mice three weeks after implantation. **(K)** PS displacement on the outer leaflet of the cell membrane of EMT6 cells or with the expression of sg*ATP11b*, or sg*ATP11b*/OE-*Ptdss2* by FACS analysis by anti-PS antibodies. **(L)** Quantification for panel **(K)** (*n* = 3 times). **(M-P)** Representative lung images from the nude mice three weeks after fat pad implantation of 628 parental cells **(M)**, 628 cells expressing *Ptdss2*-R235S **(N)**, 628 cells expressing sg*ATP11b*/OE-*Ptdss2*-628 **(O)**, and 628 cells expressing sg*ATP11b*/OE-*Ptdss2*/OE-*Ptdss2*-R235S **(P)**. **(Q)** Quantification of GFP metastatic nodules in panels **(M, N, O, P)** (*n* = 8 mice/group). Statistical data were analyzed by 1-way ANOVA with Bonferroni’s multiple-comparison test, data were presented as mean ± SEM. ***$P < 0.001$.

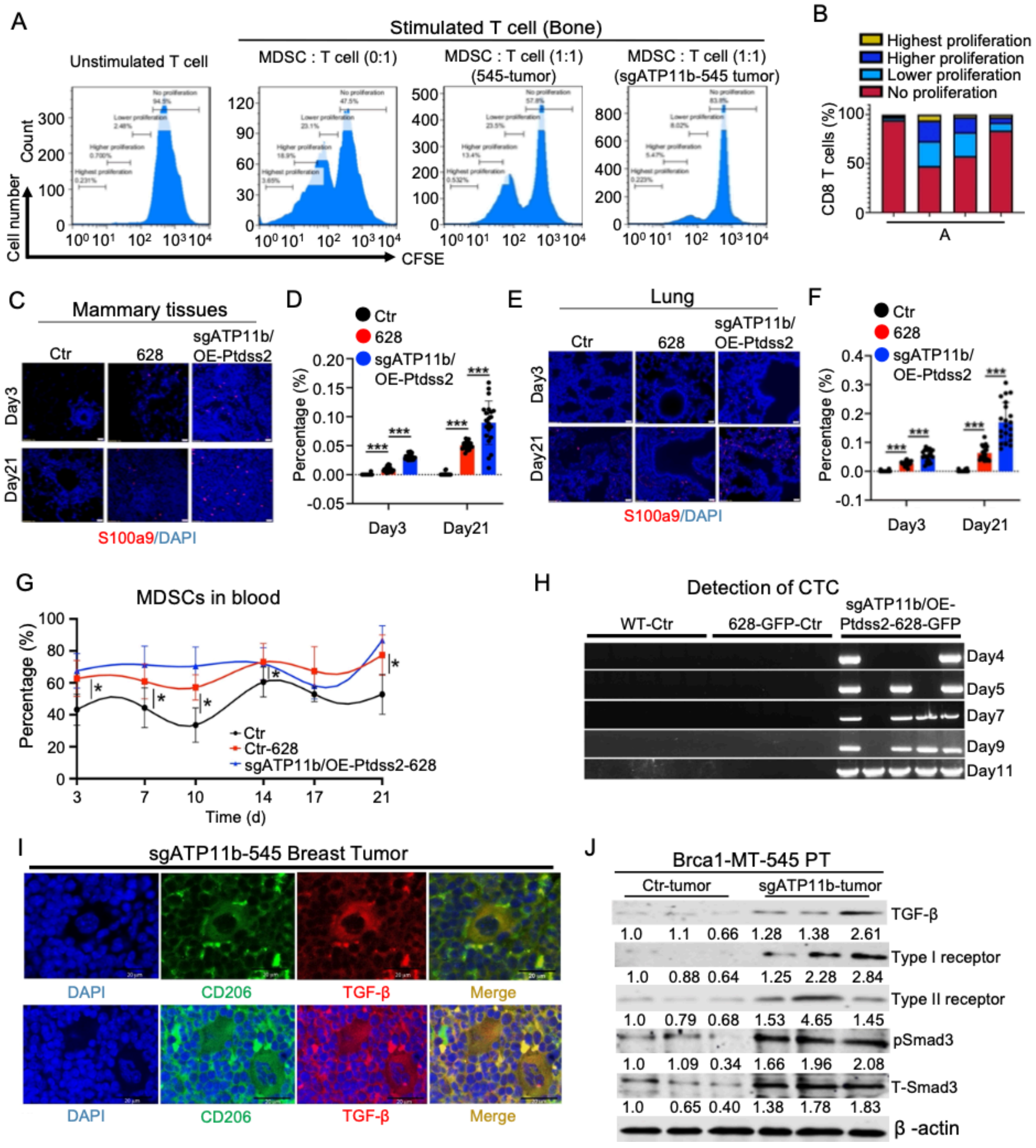

Figure S6. TME contributed by cells with high ATP11b^{low}/Ptdss2^{high} expression in vivo.

(A-B) Representative CFSE flow cytometry histograms showing the inhibition of MDSCs isolated from the bone marrow of tumor-bearing mice implanted with 545 cells without or with sg*ATP11b* expression on the proliferation of T cells stimulated with the anti-CD3/anti-CD28 antibody obtained from WT mice (A) and the summarized results **(B)** for panel **(A)** (*n* = 3 mice/group). **(C-D)** Representative IF images of mammary tissues day3 and day 21 after fat pad implantation without or

with 628 or 628 cell expressing sg*ATP11b*/OE-*Ptdss2* with S100A9 antibody staining **(C)** and quantification **(D)** for panel **(C)** *(n* = 5 mice/group). **(E-F)** Representative IF images of lung tissues day3 and day 21 after fat pad implantation without or with 628 or 628 cell expressing sg*ATP11b*/OE-*Ptdss2* with S100A9 antibody staining **(E)** and quantification **(F)** for panel **(E)** *(n* = 5 mice/group). **(G)** The accumulation of PMN-MDSCs in in blood at different time point by FACS analysis with antibodies of CD11B/Ly6G (*n* = 5 mice/group). **(H)** Detection of CTCs in blood of Ctr-WT, Ctr-628, and sg*ATP11b*/OE-*Ptdss2*-628 in nude mice as determined by PCR with luc-GFP primers for parental cells and sg*ATP11b* primer for sg*ATP11b*/OE-*Ptdss2* mice (*n* = 5 mice/group). **(I)** Co-staining of CD206 with anti-TGF-β and CD163 with anti-TGF-β antibodies on tumor tissues from sg*ATP11b*-545 mice (63X confocal microscopy) *(n* = 5 mice/group). **(J)** Protein levels of TGF-β, TGF-β Type I receptor, TGF-β Type II receptor, and pSMAD3 from primary tumors of 545-Ctr and sg*ATP11b*-545 mouse models by Western blots. Statistical data in **D, F** were analyzed by 1-way ANOVA with Bonferroni's multiple-comparison test, **G** were analyzed by two-tailed student's t test, data were presented as mean ± SEM. *$P < 0.05$, ***$P < 0.001$.

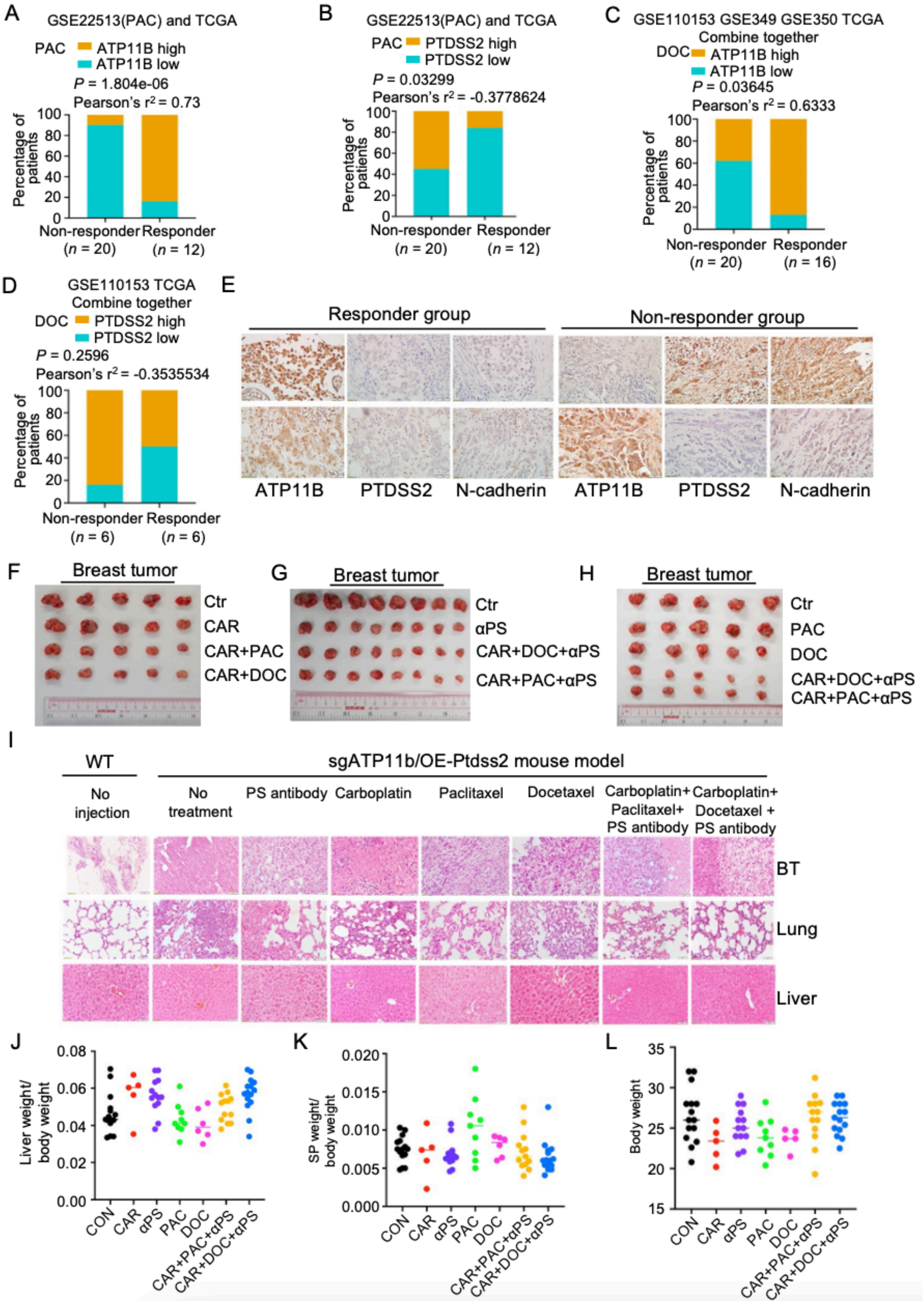

Figure S7. The effects of drug treatment in the sg*ATP11b*/OE-*Ptdss2* mouse model.

(A) *ATP11B* gene expression in clinical non-responders (n = 20 patients) and complete response responders (n = 12 patients) to paclitaxel (PAC) single treatment from the TCGA and NCBI-GEO databases. **(B)** *PTDSS2* gene expression in clinical non-responders (n = 20) and responders (n = 12) to PAC single treatment from the TCGA and NCBI-GEO databases. **(C)** *ATP11B* gene expression in clinical non-responders (n = 20) and responders (n = 16) to docetaxel (DOC) single treatment from the TCGA and NCBI-GEO databases. **(D)** *PTDSS2* gene expression in clinical non-responders (n = 6) and responders (n = 6) to DOC single treatment from the TCGA and NCBI-GEO databases. **(E)** IHC staining with ATP11B, PTDSS2, and N-cadherin antibodies in sections from a cohort of breast patients treated with docetaxel (DOC), including non-responders (n = 12 patients) and responders (n = 14 patients). **(F-H)** Representative images of primary tumors from sg*ATP11b*/OE-*Ptdss2*-628 Ctr mice (n = 13) and sg*ATP11b*/OE-*Ptdss2*-628 mice treated with CAR (n = 5), anti-PS antibody (n = 13), DOC (n = 6), PAC (n = 9), CAR+PAC (n = 5), CAR+DOC (n = 5), and combination treatment with CAR+PAC+aPS (n = 13) and CAR+DOC+aPS (n = 15). **(I)** H & E sections of mammary tumor (BT), lung, and liver tissues from normal nude mice and sg*ATP11b*/OE-*Ptdss2* mice (no treatment group) (n = 3 mice) and the treatment groups with aPS antibody, CAR, PAC, DOC, CAR+PAC+aPS (n = 3 mice), and CAR+DOC+aPS (n = 3 mice/group). **(J-L)** Plots of the body weight **(J)**, spleen **(K)**, and liver **(L)** of sg*ATP11b*/OE-*Ptdss2* Ctr mice and sg*ATP11b*/OE-*Ptdss2* mice treated with an anti-PS antibody, CAR, PAC, DOC, CAR+PAC+aPS, or CAR+DOC+aPS (n = 5-15/group). Pearson correlation test was used to calculate significance.

SUPPLEMENTAL METHODS

Mice. Five- to seven-week-old nude female mice were used for mammary fat pad implantation experiments with 1x10^6 cells injected per fat pad, and two-month-old genetically engineered *Brca1*-MSK mice were used for mammary intraductal injection. The lentiviral particles were precipitated at 3000 rpm for 30 min after incubation overnight at 4 °C in a mixture of virus supernatant with PEG-it virus precipitation solution (System Biosciences, Cat# LV825A-1) at a volume ratio of 4 to 1, and 50 µl of lentiviral particles expressing sg*ATP11b* or a mixture of viruses expressing sg*ATP11b*/OE-*Ptdss2* were injected. Tumor samples were collected five months later. DNA from *Ptdss2*-GFP-positive cells was further genotyped with specific sg*ATP11b* primers and Sanger sequencing. For the drug treatment, mice weight 20-22 g, age range from 6 to 8 months were selected, then intraperitoneal injected with PS antibody, which Synthesized by YOUKE BIOTECH company from Shanghai, category number is 3089-002 McAb and 3089-03 McAb at day 7 and day 14 (200 µg per mouse). Also, mice were intraperitoneal injected with 5 mg/kg of Carboplatin (Selleckchem, Cat# S1215), Paclitaxel (Selleckchem, Cat# S1150) or Docetaxel (Selleckchem, Cat# S1148). All mice were housed under SPF conditions in the animal facility of the University of Macau and were randomly allocated to experiments. All animal experiments were conducted under UMARE protocols approved by the Animal Research Ethics Committee of the University of Macau.

Preparation of Samples for NGS Sequencing. Genomic DNA from cells (before injection), primary tumors, blood (20 µl obtained from tail vein), recurrent and metastatic tumors was sequenced after two rounds of PCR amplification. sgRNAs were first amplified by qPCR with Phusion Flash High Fidelity Master Mix from

Thermo Fisher Scientific. The primers for the first round were: forward, 5'-CCCGAGGGGACCCAGAGAG-3', and reverse, 5'-GCGCACCGTGGGCTTGTAC-3'. The thermocycling parameters were 98 °C for 30 s, 18-24 cycles of 98 °C for 1 s, 62 °C for 5 s, 72 °C for 35 s, and 72 °C for 1 min. The barcode and index for the sequences were added to the products from the first round of PCR amplification. To ensure 80X coverage, for cell samples, five million cells were used, for tumor samples, 100 μg of DNA for each sample was used. PCR fragment was then applied for the second round of PCR according to the instructions from Illumina. The NGS was performed in The HiSeq X Ten System (Illumina company). The sgRNA reads were analyzed by MAGeCK-VISPR software (X. Shirley Liu Lab). The enriched sgRNA profile in metastatic tumors was calculated by the ratio of metastatic tumors to primary tumors. The top 20 enriched sgRNAs in different metastatic organs were considered common metastatic genes, and sgRNAs enriched in a single organ were considered organ-specific sgRNAs. The expression of sgRNA was determined by OncoPrint (Oncoplot) (https://github.com/jokergoo/ComplexHeatmap).

Detection of tumor cell DNA from the blood. 500 μL blood was extracted from the heart of mice at various days (from day 4 today 11) post implantation of tumor cells into the mammary fat pad of nude mice. The samples were diluted in 5mL of PBS containing 1.4 g/L EDTA (Ethylenediaminetetraacetic acid), then the RBCs (Red Blood Cells) were lysed by RBC lysis buffer (Cat# 00433357, Invitrogen company), and cells were washed by PBS and collected by centrifugation (G). DNA was isolated for PCR analysis using primers that are specific for tumor cells.

Construction of different genotypes. Gene knockout: Oligo sequences of candidate genes were cloned into the Lenti-V2 (vector 2), and the individual oligo sequence for sgRNA was packaged. Then the lenti-V2 vector carried sgRNA was packaged as lenti-virus and infect different cell lines, cells were selected by 4 μg/mL puromycin for 7 days, and created single clone. Finally, the change of DNA sequence was identified by Sanger sequence and viewed by SnapGene Viewer(1). Gene overexpression and point mutation: Firstly, CDS (Coding Sequence) of gene was cloned into pCDH-EF1-copGFP-T2A-Puro (Addgene, Car# 72263) vector, for the point mutation, fragment 1(from start point to mutation point) was synthesized by full length vector-Forward(such as *ATP11b* full length-Forward), point mutation Reverse (such as *ATP11b*-E186K-Reverse), and fragment 2 (mutation point to start point) was synthesized by full length vector-Reverse, point mutation Forward, then these 2 fragments were fused by overlap PCR, after picking up the desired fragment by electrophoresis, DNA fragment was cloned into pCDH-EF1-copGFP-T2A-Puro vector, finally the vector was validated by Sanger sequence.

Primers. For primers used for the qPCR and gene clone, see the table at the end of Supplemental Methods.

Western Blot Analysis. Samples were lysed in RIPA (Radioimmunoprecipitation assay buffer) buffer supplemented with protease and phosphatase inhibitors (Roche, Cat# 4906845001), and the concentration was determined by a BCA protein quantitative kit, and then, the samples were mixed with loading buffer. The samples with loading buffer were loaded onto SDS polyacrylamide gels for electrophoresis. The proteins were then transferred onto PVDF membranes,

washed, and blocked with 5 % BSA for 2 hours at room temperature. The membrane was incubated overnight with primary antibodies at 4 °C and the respective secondary antibody for 30 min at room temperature. The protein levels were determined by an ODYSSEY CXL system, and ImageJ software was used to detect the intensity of the bands.

Immunohistochemistry (IHC). Slides from Paraffin-embedded samples of breast cancer patients were collected from the Department of Pathology, the Second Affiliated Hospital, School of Medicine, Zhejiang University. Paraffin-embedded samples were sectioned at a 4-5 μm thickness. After rehydration of the tissues on slides, antigen retrieval was carried out in Antigen Unmasking R-buffer by a pressure cooker set at 95 °C for 30 min (Electron Microscopy Sciences, Cat# 62706-10). The sections were then blocked with PBS containing 1.5 % bovine serum albumin and 50% 1X animal-free blocker (Vector Laboratory, Inc. Cat# SP5030) for 1 hour at room temperature. The following primary antibodies were used: rabbit anti-TGF-β1 (Abcam, Cat# Ab92486) and rabbit anti-phosphatidylserine (MERCK company, Cat# 05-719). The sections were incubated with a mouse on rodent HPR-polymer, rabbit on rodent HPR-polymer, or rat on rodent HPR-polymer for 1 hours. All the reagents were included in the Histostain-Plus IHC kit, and all the polymers were broad spectrum HRP-polymers (Invitrogen, Cat#859043) and then developed with a DAB Plus substrate detection system (Biocompare, Cat# SK-4100) for 5-10 min at RT, counterstained with hematoxylin, and dehydrated. After a cover slip with Permount was added, the IHC slides were viewed with Olympus BX53.

Immunofluorescence. Cells (5x10^4 or 1x10^5) were seeded in 24-well plates was a circle cover glass (Thermo Fisher Scientific, Cat# 12CIR-1) and cultured overnight. The cells were then fixed with freshly made PBS containing 4% formaldehyde solution (w/v) and methanol-free formaldehyde (Thermo Scientific, Cat# 28906) at room temperature for 30 min and blocked with PBS containing 1.5 % bovine serum albumin and 50% 1X animal-free blocker (Vector Laboratory, Inc. Cat# SP5030) for 1 hour at room temperature. The following antibodies were used: rabbit anti-TGF-β (Abcam, Cat# Ab92486), anti-ATP11B (Abcam, Cat# Ab189392), anti-PTDSS2 (LSBio, Cat# LS-C117251-100), mouse anti-CD163 (Abcam, Cat#Ab182422), CD206 (Abcam, Cat# Ab64693). and anti-E-cadherin (BD, Cat#610182). For double staining, the slides were incubated overnight with a mixture of two primary antibodies at 4°C in blocker solution. The slides were then washed with cold PBS, and a mixture of secondary antibodies was used: Alexa Fluor 488 goat anti-mouse (Life Technologies, Cat# A21121) and Alexa Fluor 594 labeled goat anti-rabbit (Life Technologies, Cat# A27016). The slides were then counterstained with DAPI/prolonged diamond antifade mountant (Cat#. P36961) and examined by a ZEISS LSM 710 confocal microscope and a ZEISS LSM 880 high-resolution microscope with Airyscan.

RNA-seq. Mammary gland tissues, primary tumor tissues, tumor adjacent tissues, and metastatic tumor tissues were homogenized by a Precellys Evolution tissue homogenizer. Extracted RNA sampleas were examined by Agilent RNA kits for use with the 2100 Bioanalyzer System and sequenced by NGS (HiSeq 2500 platform). The fastq file quality was checked by fastqc software, and the reads were aligned by HISAT2 software (based on the grcm38 reference sequence). The

expression values were determined by StringTie software. The FPKM-based RNA-seq differentially expressed genes were extracted by Ballgown software, and pathways enriched with differentially expressed genes were analyzed by the “clusterProfiler”(https://bioconductor.org/packages/release/bioc/html/clusterProfiler.html) package in R.

T Cell Suppression Assay. MDSCs were isolated from primary tumor tissue four weeks after implantation of 628-GFP cells expressing sg*ATP11b*/OE-*Ptdss2*. The tumor tissues were first digested with 5 ml of digestion I buffer (5 mg/ml insulin, 5 mg/ml HC, 10 ng/ml EGF, 300U/ml collagenase III, 100 U/ml hyaluronidase, and 20 ng/ml cholera toxin) for one hour in 37°C. After centrifugation at 3000 rpm for 5 min, the cells were treated with RBC lysis buffer (Invitrogen, Cat# 2155206) to lyse the blood cells. Single cell populations were then labeled with CD11B and Gr1 antibodies and stored on ice for 45 min, and MDSC cell populations were sorted by a FACS Aria II flow cytometer. T cells isolated from WT spleen and bone marrow were labeled with CFSE (Invitrogen, Cat# C34554) and mixed with anti-CD3 (Santa, Cat#SC-20047) with anti-CD28 (BioLegend, Cat# 100204) antibodies. T cells were then mixed with MDSCs at a ratio of 0:1 or a 1:1 with a total of 5.0x105 cells in each well (24-well plate). T cell proliferation was determined by FACS analysis after the cells were cultured for 96 hours.

Mass Cytometry (CyTOF) and Flow Cytometry. Disassociated single cells were depleted of erythrocytes by RBC buffer (Invitrogen, Cat# 2155206), and three million single cells from tumor tissues were suspended in 50 μl of Maxpar cell staining buffer. The cells were blocked with Fc receptor blocking reagent (Miltenyi

Biotec, 130-059-901) at room temperature for 10 min. and incubated with CyTOF antibodies, including anti-CD11B, anti-Ly6G, and anti-Ly6C, for 30 min at room temperature. The cells were washed once and then incubated with Cell-IDTM. Cisplatin (Fluidigm, 201064) at 2.5 mM was added to cultures for 2.5 min to stain viable cells. The cells were fixed overnight with MaxparRFix and Perm Buffer containing Cell-IDTM Intercalator-Ir (Fluidigm, 201192) at 0.125 mM and 4 °C. The cells were analyzed the next day with a Helios CyTOF system in the FHS Genomic Core Facility at the University of Macau. CyTOF data were analyzed by R 3.4.3 software, and packages used in the analysis included flowCore, CATALYST, matrixStats, Rtsne, and FlowSOM. To normalize the matrix, 7000 cells were randomly selected from each sample and clustered by the Rtsne package. After clustering, the ggplot2(https://cran.r-project.org/web/packages/ggplot2/index.html) package was used to identify the expression of each marker, and then, the cells were clustered again by using the BuildSOM function in the FlowSOM package. The visualization was completed by the ggplot2 package. For the detection of cell apoptosis, it's performed by using APO-BRDU^{TM} kit(Novus Biologicals company, Cat# NBP2-31161)

Promoter Assay. The promoter sequence information of *Ptdss2* between -1499 and +100 was downloaded from the EPD website(2) and cloned into a PGL basic vector. PGL-basic vector or PGL-*Ptdss2* vector together with m*Brca1* cDNA, control DNA, and Renilla DNA were co-transfected into WT and *Brca1*-MT mammary epithelial cells in 24-well plates for 72 hours. After washing the cells with PBS twice, 100 μl of PLB solution from a luciferase assay system kit (Promega company, Cat# E1500) was added to lyse the cells. Spontaneous fluorescence

was detected by a microplate reader after adding 100 μl of LAR II and 100 ul stop&glo to each well. Quantification of signal intensity was performed by using the ratio of fluorescence intensity first time/fluorescence intensity second time.

Computational Analysis. TCGA-BRCA data was downloaded from Broad GDAC Firehose website(3). Clinical information, including survival state and follow-up information. The drug treatment information was from the Merge Clinical dataset. The mRNA expression data were obtained from the Illuminahiseq_rnaseqv2-RSEM_genes_normalized dataset. The mRNA expression data were first normalized by VOOM function in the limma package. The z value was calculated based on mRNA expression in healthy donors. Patients were divided into target genes with high expression and low expression based on the median z value. Then, the survfit function was used from the survival package to calculate the relation between gene expression and the overall survival rate. Finally, the survival curve was plotted with the survminer package.

For analysis of GEO (Gene Expression Omnibus) and other databases, the gene expression dataset of the GEO database was downloaded by GEOquery (https://www.bioconductor.org/packages/release/bioc/html/GEOquery.html) software. The GSE datasets used in this article included GSE61304, GSE21653, GSE22513, GSE110513, GSE349 and GSE350. The expression data were extracted by the Exprs function, annotated by the related GPL platform, and normalized by log2 transformation. Network analysis were performed by using RTN(https://www.bioconductor.org/packages/release/bioc/html/RTN.html) package. In addition, clinical information of patients, including follow-up time and patient survival status, was extracted by the pData function. Finally, the

relationship between gene expression and the overall survival rate was determined. For drug selection analysis, drug treatment information was first extracted by the pData function and then merged with the expression of the target gene, and the box plot was completed using the Beeswarm function of the Beeswarm package. For the figure 8 A-C, dataset was firstly downloaded by GEOquery package, then the expression value of ATP11B and PTDSS2 were normalized by normalize.quantiles function of preprocessCore package, and mean of ATP11B and PTDSS2 in all the samples were calculated. Finally, the ATP11B^{high}/PTDSS2^{high} group were determined by samples which both ATP11B and PTDSS2 expression are higher than mean of all the ATP11B expression and PTDSS2 expression. Samples which ATP11B expression is higher than mean of ATP11B, and PTDSS2 expression is lower than mean of PTDSS2 were allocated to ATP11B^{high}/PTDSS2^{low}. Sample which ATP11B expression is lower than mean of ATP11B and PTDSS2 expression is higher than mean of PTDSS2 were classified as ATP11B^{low}/PTDSS2^{high} group. All data were analyzed by R 3.4.3 software. The count data were analyzed by paired t-test and chi square test. The difference was statistically significant when the p value was lower than 0.05.

For the counting of cells in IF image. All IF images were captured by Polarizing Microscope (OLYMPUS, Cat# BX53-P) and all images were analyzed by Python 3.8 software. All the images were divided into single color by Pillow (4) package. The number of cells which had single color, in this experiment the blue (DAPI), red(S10A9), were extracted and counted by cellpose (5) and OpenCV (6) package. Based on the area and location of rectangles in blue, red, or green images, the double negative, double positive, S10A9 single positive, and CD11B single positive

rectangles were quantified, and the ratio were represented as heatmap by pheatmap package in R software.

Table. Primers used for qPCR and gene clone

Primer sequence	Forward	Reverse
qPCR primer		
Brca1 mouse	CTGAAGACTGCTCAGGGCTATC	AGGGTAGCTGTTAGAAGGCTGG
ATP11b mouse	GCCAACTTGGACAGTCTCATAGC	ATCTGGCTCCACGAAGCAAGAG
Ptdss2 mouse	CTACGACGATGGCACTAACACC	GTGTTGTAGGCTGTATCCTGAGG
Ptdss1 mouse	CAGCAAGTGGAGGACATCACCA	CCTCTCCAGATGTTGTCTTCCG
Acaca mouse	GTTCTGTTGGACAACGCCTTCAC	GGAGTCACAGAAGCAGCCCATT
Fasn mouse	CACAGTGCTCAAAGGACATGCC	CACCAGGTGTAGTGCCTTCCTC
BRCA1 human	CGAGGAAATGGCAACTTGCCTAG	TCACTCTGCGAGCAGTCTTCAG
ATP11B human	GAATGCCAGCAACCAGAAGCAG	AATCTGGCTCCACGAAGCAGGA
PTDSS2 human	CAGCGTGATGTTCGAGTTCCTG	GGTCTTCATGCCGCAGTAGATG
PTDSS1 human	CGTAGTTACGGTCTCTGCTGGA	GCCATTGCACAACAGGATGTCC

sgRNA related primer		
1st amplification primer	AATGGACTATCATATGCTTACCG T AACTTGAAAGTATTTCG	GAGCCAGTACACGACATCAC
ATP11b sgRNA(mouse)	AAACTCGATCCAAAAACATT	AATGTTTTTGGATCGAGTTT
Ptdss2 sgRNA(mouse) -1	TTGTAGCTTACTGGCGGTTT	AAACCGCCAGTAAGCTACAA
Ptdss2 sgRNA(mouse) -2	AGTCACGTAGCCCAGCGCAC	AAACCGCCAGTAAGCTACAA
Ptdss2 sgRNA(mouse) -3	GTGCGCTGGGCTACGTGACT	GAGAGGGACTACGGGGGCAA
ATP11B sgRNA(human) -1	AACCATCTACGTAGCCAAC	GTTGGCTACGTAGATGGTTC
ATP11B sgRNA(human) -2	GCCATTCTGAGGAAACCTGT	ACAGGTTTCCTCAGAATGGC
ATP11B sgRNA(human) -3	GGGAAAGATACATCTCTGCT	AGCAGAGATGTATCTTTCCC
PTDSS2 sgRNA(human) -1	GATGACCAGGGCAGCACCGT	ACGGTGCTGCCCTGGTCATC

PTDSS2 sgRNA(human)-2	CAAGTTACCTGGATGAGGTC	GACCTCATCCAGGTAACTTG
PTDSS2 sgRNA(human)-3	GCTCCGTCCTGGCGCTCACC	GGTGAGCGCCAGGACGGAGC
cDNA primer		
ATP11b-full length	CTAGCTAGCTAGATGTGGCGCTGGGTCCGGCA	CGGAATTCCGCAATGGACTCATCTACTTGT
ATP11b-E186K	ATCATTCCAATTTCTAAATATGTGACAGTTGAGATGC	ATCTCAACTGTCACATATTTAGAAATTGGAATG
Ptdss2-full length	CTAGCTAGCTAGATGCGGAGGGGCGAGC	CGGAATTCCGTCATGACGCGGCTGAGGT
Ptdss2-R235S	TGCAATCGGCTGGGCATCTACTGTGGC	ATGCCACAGTAGATGCCCAGCCGATTG

Reference

1. Sakuma T, Takenaga M, Kawabe Y, Nakamura T, Kamihira M, and Yamamoto T. Homologous Recombination-Independent Large Gene Cassette Knock-in in CHO Cells Using TALEN and MMEJ-Directed Donor Plasmids. *Int J Mol Sci.* 2015;16(10):23849-66.
2. Schmid CD, Perier R, Praz V, and Bucher P. EPD in its twentieth year: towards complete promoter coverage of selected model organisms. *Nucleic Acids Res.* 2006;34(Database issue):D82-5.
3. Cheng PF, Dummer R, and Levesque MP. Data mining The Cancer Genome Atlas in the era of precision cancer medicine. *Swiss Med Wkly.* 2015;145:w14183.
4. van der Walt S, Schonberger JL, Nunez-Iglesias J, Boulogne F, Warner JD, Yager N, et al. scikit-image: image processing in Python. *Peerj.* 2014;2.
5. Stringer C, Wang T, Michaelos M, and Pachitariu M. Cellpose: a generalist algorithm for cellular segmentation. *Nat Methods.* 2021;18(1):100-6.
6. He B, Lu Q, Lang J, Yu H, Peng C, Bing P, et al. A New Method for CTC Images Recognition Based on Machine Learning. *Front Bioeng Biotechnol.* 2020;8:897.
